# Barriers to patient, provider, and caregiver adoption and use of electronic personal health records in chronic care: a systematic review

**DOI:** 10.1186/s12911-020-01159-1

**Published:** 2020-07-08

**Authors:** Zahra Niazkhani, Esmaeel Toni, Mojgan Cheshmekaboodi, Andrew Georgiou, Habibollah Pirnejad

**Affiliations:** 1 Nephrology and Kidney Transplant Research Center, Urmia University of Medical Sciences, Urmia, Iran; 2 Department of Health Information Technology, Urmia University of Medical Sciences, Urmia, Iran; 3 Student Research Committee, Urmia University of Medical Sciences, Urmia, Iran; 4 Office for Disease Registry and Health Outcomes, Urmia University of Medical Sciences, Urmia, Iran; 5 Centre for Health Systems and Safety Research, Australian Institute of Health Innovation, Macquarie University, Sydney, Australia; 6 Patient Safety Research Center, Urmia University of Medical Sciences, Urmia, Iran; 7 Erasmus School of Health Policy & Management (ESHPM), Erasmus University Rotterdam, Rotterdam, The Netherlands

**Keywords:** Personal health records, Systematic reviews, ePHR, Self-care, Chronic diseases

## Abstract

**Background:**

Electronic personal health records (ePHRs) are defined as electronic applications through which individuals can access, manage, and share health information in a private, secure, and confidential environment. Existing evidence shows their benefits in improving outcomes, especially for chronic disease patients. However, their use has not been as widespread as expected partly due to barriers faced in their adoption and use. We aimed to identify the types of barriers to a patient, provider, and caregiver adoption/use of ePHRs and to analyze their extent in chronic disease care.

**Methods:**

A systematic search in Medline, PubMed, Science Direct, Cumulative Index to Nursing and Allied Health Literature (CINAHL), the Cochrane Central Register of Controlled Trials, and the Institute of Electrical and Electronics Engineers (IEEE) database was performed to find original studies assessing barriers to ePHR adoption/use in chronic care until the end of 2018. Two researchers independently screened and extracted data. We used the PHR adoption model and the Unified Theory of Acceptance and Use of Technology to analyze the results. The Mixed Methods Appraisal Tool (MMAT) version 2018 was used to assess the quality of evidence in the included studies.

**Results:**

Sixty publications met our inclusion criteria. Issues found hindering ePHR adoption/use in chronic disease care were associated with demographic factors (e.g., patient age and gender) along with key variables related to health status, computer literacy, preferences for direct communication, and patient’s strategy for coping with a chronic condition; as well as factors related to medical practice/environment (e.g., providers’ lack of interest or resistance to adopting ePHRs due to workload, lack of reimbursement, and lack of user training); technological (e.g., concerns over privacy and security, interoperability with electronic health record systems, and lack of customized features for chronic conditions); and chronic disease characteristics (e.g., multiplicities of co-morbid conditions, settings, and providers involved in chronic care).

**Conclusions:**

ePHRs can be meaningfully used in chronic disease care if they are implemented as a component of comprehensive care models specifically developed for this care. Our results provide insight into hurdles and barriers mitigating ePHR adoption/use in chronic disease care. A deeper understating of the interplay between these barriers will provide opportunities that can lead to an enhanced ePHR adoption/use.

## Highlights

- Evidence points to benefits associated with PHR adoption and uses in chronic conditions
- Barriers to PHR adoption/use, with a special focus in chronic care, has not been well described and understood
- Addressing barriers for PHR adoption/use in chronic care should cross the boundary of patient-level barriers
- Barriers at the provider and healthcare organization levels should be understood and addressed, thoroughly
- PHRs should fit in the structure of “chronic care models” developed for improving chronic care

## Background

Promoting self-care and patient engagement in care management has gradually become key features in efforts to improve health service delivery and care quality in chronic diseases [1, 2]. Electronic personal health records (ePHRs) provide the tools to empower patients and promote self-care [3, 4]. A systematic review found that self-monitoring through ePHR improves health outcomes in chronic conditions [5]. Because of such potentials to enhance quality and patient engagement [6–8], the Health Information Technology for Economic and Clinical Health Act (HITECH) and meaningful use phase 2 and 3 have driven the adoption of ePHRs in parallel to Electronic Health Records (EHRs) [9].

Studies have shown that both patients and providers are interested in ePHRs especially as they find them as a means to increase patient empowerment [10–12]. Yet, there are barriers to overcome and challenges to embrace when adopting ePHRs. Some of these barriers are related to the implementation of EHRs such as EHR products and capital and human resource issues. For example, from 2,674 general hospitals studied in the United States (US) in 2013, only 5.8 percent of hospitals met measures for stage 2 meaningful-use readiness and several other criteria, including sharing care summaries with other providers and providing patients with online access to their data, as necessary functions for a tethered PHR [13]. Other barriers are more ePHR specific ones such as poorly aligned functionalities with patients’ expectations and self-management practices and concerns about privacy and confidentiality of patient information in ePHRs [14, 15]. Even outside the US healthcare context, similar hurdles have also contributed to a lower adoption rate than what has been expected or hoped for [16]. Such results continue to be reported after the implementation of many health information technologies (HIT) including ePHRs, which highlight a strong need to understand factors and challenges that influence the implementation outcomes [17]. Overcoming these challenges and barriers in implementing and adopting ePHRs can result in increased efficiency and improved quality patient care [18]. Therefore, recognizing and understanding the nature of such barriers is imperative to be well equipped to devise strategies to overcome the barriers and to achieve ePHR’s meaningful use.

There have been a few reviews published on the barriers to ePHR adoption and use. A review of the *patient-level* barriers categorized them into individual, demographic, capability, health-related, ePHR-related, or attitudinal factors [19]. Another review with similar scope concluded that a lack of awareness of and sufficient training regarding portal use were the two main barriers [18]. In the elderly population, the main barriers were limited technology access and no prior knowledge of the existence of a patient portal, and limited health literacy and motivation to use a patient portal [20]. In rural areas of the US, provider resistance, privacy concerns, and the lack of EHRs, interoperability standards, and funding have emerged as the main barriers [21]. However, these reviews have narrowly focused on *patient-level* barriers [18, 19], or were limited in terms of age ranges [20], time frame, or geographical location reviewed [21, 22]. To our knowledge, there is a significant gap in the literature on the barriers in the patient, caregiver, and provider levels that may impact ePHR adoption and use in the context of *chronic care*. To address this gap, we aimed to identify and synthesize evidence on ePHR adoption and use barriers in chronic disease care. More specifically, we were interested to identify the types of barriers and to analyze their extent in this care. The insights gained will inform efforts for effective design, implementation, and use of ePHRs for a patient population at the most need of these tools.

## Methods

This review was conducted according to the Preferred Reporting Items for Systematic reviews and Meta-Analyses (PRISMA) [23].

### Search strategy

We conducted a literature search in OVID versions of MEDLINE, PubMed, Science Direct, Cumulative Index to Nursing and Allied Health Literature (CINAHL), the Cochrane Central Register of Controlled Trials and the IEEE database for English-language, journal or congress proceedings’ full texts published January 1, 2005, till December 31, 2018. We used a Boolean search strategy using keywords and MeSH terms related to two areas of interest i.e., the intervention (e.g., Personal Health Record OR Personal Medical Record OR patient portal OR patient internet portal, etc.) *AND* the health condition (e.g., Chronic Disease OR Chronic Illness, OR Chronic Condition, etc.). The details of our search strategy are accessible in Additional file 1. We also conducted a manual review of all reference lists of included studies and the pertinent ePHR reviews including [14, 15, 18–22, 24–29].

### Inclusion and exclusion criteria

We included studies according to the following inclusion criteria: 1) the intervention was an ePHR/patient portal, 2) the targeted users were *chronic* disease patients, their caregivers and/or their healthcare professionals, 3) the study was an original research article, and 4) the study design was either quantitative, qualitative, or mixed methods.

We excluded ePHR/patient portals that were not aimed at chronic patients, paper-based ePHRs or educational websites, assistive living technologies, or mHealth tools, systematic reviews, proceedings abstracts, commentaries, editorials, and articles describing theoretical background or design reports without having an evaluation nature. The main reasons for exclusions in each phase of this review are accessible in Additional file 2.

### Review procedures and data extraction

**Fig. 1:**
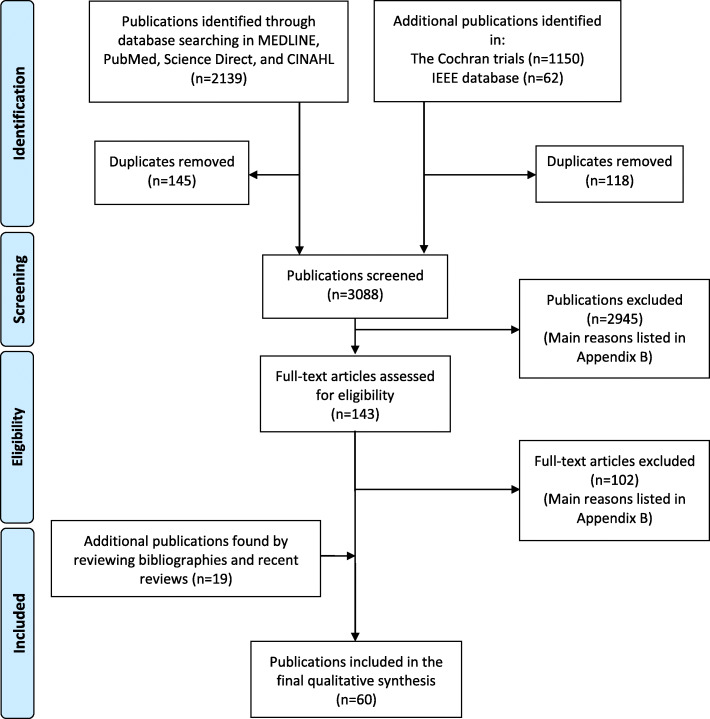
Flow diagram of study selection (literature search January 1, 2005 till December 31, 2018)

After removing duplicates, our search identified 3088 unique records, which were screened for eligibility. Figure 1 shows the PRISMA flow diagram of our review. Two reviewers (ET and MCH) were trained on the screening and data extraction tool by ZN, who is an experienced researcher in conducting systematic reviews in the field. The reviewers reviewed a sample of references and compared extraction results to reach an excellent agreement (kappa= 0.77). Then, they screened titles and abstracts of the above-mentioned search result to find relevant studies based on our inclusion/exclusion criteria. In this phase, 143 potentially eligible publications were selected for the full-text review. Further articles were found through the manual review. All articles were independently reviewed in detail by ZN and either ET or MCH. Disagreements were solved by consensus. Endnote version XI was used to manage records.

We extracted the following main study characteristics in the full review phase: general information (e.g., the authors and publication year), study objectives, study design, patient population, system users, the intervention (e.g., the description of ePHRs and their integration with other systems), and the main study results.

### The methodological quality of studies

We used the mixed methods appraisal tool (MMAT) version 2018 to assess the quality of evidence in included studies [30]. This tool can be used to appraise the quality of empirical studies (i.e., primary research based on experiment, observation, or simulation) in three categories of study designs (i.e., qualitative, quantitative, and mixed methods).

### Data synthesis

According to a widely used definition, an ePHR is “an electronic application through which individuals can access, manage and share their health information, and that of others for whom they are authorized, in a private, secure, and confidential environment” [3]. We used two well-known models as a theoretical background to analyze and categorize barriers to ePHR adoption/use faced by users. The first was the “Personal Health Records Adoption Model” (PHRAM), developed through integrating several relevant parent models/theories [31]. This model was used to analyze the barriers faced by patients and caregivers in the context of chronic care. We also used the unified theory of acceptance and use of technology (UTAUT) to analyze barriers specifically experienced by care providers [32]. Since conducting a meta-analysis became out of the scope of this study due to the lack of unified quantitative data in included studies, we only provide a narrative description of results based on the PHRAM and UTAUT.

## Results

### Characteristics of included studies

Our review identified 60 ePHR studies [5, 10, 12, 33–89], among which 24 were qualitative, 22 quantitative, and 14 mixed methods studies. Additional file 4 provides the details of the included studies. These studies were conducted between 2006 and 2018, nine of them in the single year of 2015. Forty-six studies were from the US, followed by five studies in Canada, two studies in the Netherlands, two in the United Kingdom, and the remaining five in Denmark, Sweden, Germany, New Zealand, and Argentina (one study from each country). A majority of studies included older patient populations (compared with younger patients) and diabetics (compared to other chronic patients) in their evaluations. Ten studies had a target population of pediatrics [37–39, 43, 44, 59, 71, 72, 78, 85]. The results are provided here according to the personal, environmental/medical practice, technological, and chronic disease factors on the bases of the PHRAM and UTAUT.

### Personal factors

In one study involving survivors of pediatric cancers, referring to the issue of age, cancer survivors >18 years old were significantly more likely to use an ePHR compared to those <18 [85]. While a high proportion of patients with age 50 and older had higher frequency and intensity of ePHR use [66], patients over the age of 65 were less likely to intend to use an ePHR [10], and patients aged over 70 were associated with a lack of use [70]. In four studies, more men than women had access to computers or the internet, expressed confidence in using ePHRs, or used it [54, 67, 74, 84], while females were the dominant users in three studies [48, 66, 89].

People with minority race/ethnicity (including African-American, Latino, and Filipino) reported more negative attitudes towards ePHRs, were less likely to use ePHRs and experienced more obstacles compared to Whites and Caucasians [34, 47, 48, 55, 57, 61, 65, 66, 69, 70, 74, 75, 85]. Having a paid job or higher income and living in a higher socioeconomic neighborhood, and being insured were associated with higher use (reported in studies from the US and the Netherlands) [42, 61, 66, 67, 75, 84]; while, having a lower income and being below the poverty level were linked to non-use [34, 48].

Patients with lower educational attainments were less likely to intend to use or use an ePHR [10, 34, 42, 48, 61, 65, 67, 69, 70, 74, 75]. Patients with limited health literacy were less likely to use ePHR or use it ineffectively [5, 34, 41, 46, 54, 65, 69, 74, 76, 77, 87]. The level of knowledge, self-efficacy, and confidence in technology use was associated with ePHR adoption/use [52, 54, 56, 67, 83].

Negative attitudes toward the disease and health care experiences in general, and ePHRs in particular, prevented patients from using ePHRs [10, 40, 44, 47, 59, 71]. Patients were concerned about the reliability of ePHRs to facilitate timely and productive communication with providers [37, 43, 65, 81]. In one study, patients commonly expressed negative attitudes partly because of their experience of confusion and misunderstanding [40].

Fourteen studies highlighted the critical role of computer/technology literacy and skills to effectively use ePHRs [5, 41, 42, 45, 49, 52, 56, 65, 68, 76, 82, 83, 86, 87]. Computer literacy barriers included, but were not limited to, the lack of basic computer skills, inexperience in using search bars or uniform resource locators, difficulty while navigating the portal, and negative experiences with online security breaches/viruses. Three studies noted that computer anxiety negatively affected patients’ behavioral intention to adopt ePHRs [5, 34, 86].

Challenges related to communication preferences were brought up in several studies with a majority pointing out the value of in-person, or telephone contacts between patients and providers [37–40, 44, 47–49, 52, 58, 59, 64, 65, 76, 78–80, 82, 86]. The main reasons for such a preference were getting anxious when seeing results online and concerns over technology replacing their providers. The preference for in-person communications was also shared by providers in certain circumstances [64, 80].

### Environmental/medical practice factors

#### Social influence

The impact of the social influence of “important others” (i.e., family members and care providers) on patients was evident [12, 54, 55]. It was shown that living alone and being not currently married were associated with non-adoption and lesser ePHR use [34, 42, 67]. Moreover, studies pointed out the role of providers’ willingness to use portals, their communication about it, and their level of use in patients’ initial portal use [47, 59, 81, 84]. While patients wanted their physicians to get more involved in ePHRs [79], physicians viewed them as more of a patient, receptionist, or nurse tool [68, 79].

#### Facilitating/impending conditions

Our review identified the existence or otherwise lack of the following organizational and/or technical infrastructures to support or impede ePHR use: being in an organization’s priority list, integration into the EHRs, patients ready access to resources such as computers, the Internet, and ePHRs, adequate technical support, and proper training on ePHR use [5, 10, 12, 34, 42, 46, 50–52, 56, 60, 64, 65, 76, 84, 88].

Due to its impacts on physician’s time management and workload, “physician resistance” was mentioned as “the greatest barrier to ePHR implementation” [12]. There were also concerns about the impacts on providers’ available time for care, lack of reimbursement, or professional liability issues [36, 64, 68]. Physicians voiced their concerns about excess time and efforts to handle issues related to the ePHRs due to lack of integration with EHRs [79, 80].

#### Incentive motivation

Tangible incentives and cost compensations, or otherwise lack thereof, were also an important factor [12, 54, 64, 65]. For example, it was important to be certain about how ePHR-related services would be paid for, who would pay, and under what circumstances [41]. The cost of services was also mentioned as a barrier by patients [76, 83, 88].

### Technology factors

This section provides the results related to the perceived usefulness of ePHRs, perception of external control, compatibility, and perceived complexity.

#### Perceived usefulness

Perceived usefulness featured as a key driving factor for the intention to use ePHRs [10, 49, 59, 65, 79, 80]. For example, non-users mostly expressed concerns about simply not seeing the value of using a portal to manage their health or lack of personalization in using this technology [65].

#### Perception of external control

Preserving general privacy, confidentiality, and security of health records was one of the most common concerns regarding ePHR use (e.g., confidentially of a stigmatized or sensitive condition, or confidentiality and security of information easily accessible to researchers and industry members, and misuse of information by insurance companies to deny coverage) [10, 45–47, 52, 58, 65, 68, 72, 76, 78, 87, 88]. Patients voiced their concerns about caregiver’s access to their information and requested appropriate access limitation [52, 53, 68]. Clinicians’ attitudes towards caregiver involvement in ePHR use were controversial in one study: while 28.3% favored it, 32.1% disagreed because it impaired patients’ privacy [80].

Moreover, patients reported frustration at several instances in which their profile, medication list, lab results or medical history were incorrect or missing in the ePHR but they were unable to correct them [46, 50, 64]

#### Compatibility

The degree to which an ePHR was perceived as being consistent with the existing values, past experiences, and needs of its potential adopters i.e., chronic patients and their caregivers and providers were mentioned as an important factor for adoption in some studies [36, 40, 44, 64, 90]. When comparing with the traditional chronic care, users asked for much easier navigation through ePHRs, access to additional information (e.g., progress notes, outside test results, personalized medication information, and a structure to track the course of treatment) or a customized ePHR based on their specific chronic illness [36, 39, 41, 44, 46, 50, 60, 62, 78, 80].

#### Perceived complexity

The difficulty of understanding or navigating an ePHR was one of the most common barriers referred to in the included studies. Use of problematic medical jargon, confusing information display, and unclear presentation of information based on patients’ knowledge (e.g., unclear numeral values and unfamiliar medical terms) were only some of the barriers that prevented effective ePHR use [33, 35, 38–41, 46, 49, 50, 59, 60, 68, 76, 78, 79, 82].

### Characteristics of chronic disease

#### Attitudes on negotiated collaboration and preferences for self-regulation

On the one hand, the feeling of having more control over the disease was a reason for limited portal use by patients [34, 59, 79]. Providers also doubted whether patients who were proficient at monitoring their disease were the right group to benefit from ePHRs [79]. On the other hand, being an active healthcare consumer and having a worse or higher proportion of co-morbid conditions and taking more prescribed medications were linked to ePHR use [34, 45, 54, 66, 84, 89]. It was also noted that patients’ willingness to take responsibility for their health through ePHR depended on their coping style and perceived competence and autonomy [71]. In a study, patients who “felt too confronted when monitoring the course of their illness” dropped out of an ePHR [80].

#### The perceived complexity of care

Based on the task-technology-fit model, a study found instances of mismatches between user mental models and the technology, which manifested primarily as vocabulary misunderstandings, as portal functionality that did not perform as the patient expected, and as requests for clarification and help [33]. They did not have a very concrete understanding of how health information management tasks and processes underlying the ePHR worked.

#### Characteristics of healthcare settings, providers, and chronic illnesses

In one study, patients in rural settings were less likely to use ePHRs compared with those in urban settings [34]. However, if patients received care at multiple sites, they were more likely to use ePHRs. Patients acknowledged the need to consolidate data produced by multiple providers and scattered in different locations through ePHRs [33]. A lack of interoperability between ePHRs and EHRs in provider offices was noted in three studies resulting in excess workload and frustration [46, 50, 80]. In a survey of patients from 29 states across the US, with at least 38 different types of portals, 51% reported having two or more portal accounts creating frustration when it came to patients remembering their names, and managing different portals from different providers [56]. This was a concern in another study, too [65].

Another problem was confusion over who should receive and reply to messages on the provider team i.e., a physician, a nurse, the office staff, or the entire care team; because this would impact the content of patient messages [37, 46, 86]. In another study, patients had unsatisfactory communications with the care team through a portal; for example, they failed to track their health issues in a coherent way [43]. Physicians were concerned about clarity about responsibilities (and potential liabilities) related to responding to patient-added information or commentaries seen by several different clinicians [36]. For the sake of clarity, Table 1 provides a summary of provider-specific barriers.

**Table 1:** Barriers to the adoption and use of care providers on the basis of the UTAUT

Authors (year of publication) ^**b**^	Study objective	Research method	Country	Features of PHRs (if any)	Integration with EMR/EHR	PHR's target chronic patient population	Study participants	Main outcomes in relation to the objective of the current study
Lober et al. (2006) [5]	To evaluate the barriers faced by a low income, elderly population in creating and using a personal health record.	Qualitative	The USA	A Personal Health Information Management System (PHIMS), allows viewing personal demographics, past surgeries and immunization records, environmental factors and foods, medications and allergies to medications, also with capabilities of messaging with provider, sharing printed version of information with providers or family	Untethered	Adults elderly patients	38 elderly residents of a nursing home, many had chronic disease	- Health and computer literacy and anxiety - Physical and cognitive impairments of elderly - Problems related to access to the computers (e.g., not owning a computer) and access to an assistant to use the system (e.g., availability of nurses or social workers)
Hess et al. (2007) [50]	To explore challenges to office-based implementation of a patient portal and initial patient reaction to the technology in the context of diabetes care	Qualitative	The USA	University of Pittsburgh Medical Center (UPMC) HealthTrak (a patient portal) allowing to view test results, medication and problem lists, and health reminders, secure, electronic communication with the physician’s office, to view and schedule appointments, and disease-specific tools and information plus self-management tools for weight and blood pressure monitoring	Tethered	Adults diabetic patients	Diabetic patients	- Patient identified inefficiencies including missing lab results and radiology reports, inaccurate information, and slow responses from the physician and/or nurse. - Barriers to use, including lost or unknown user names and passwords, being unaware of the features of the HealthTrak, not possible to contact all of patients’ physicians, not just their primary care physician, due to lack of coordination and integration
Zickmund et al. (2008) [86]	To discern the impact of the provider–patient relationship on interest in using a web-based patient portal	Qualitative	The USA	“HealthTrak”, a patient portal originally offered online information, laboratory results, and an encrypted and secure method for e-mailing messages. The enhanced version for diabetes patients allowed them to track glucose, blood pressure, and physical activity records online entered by them	Tethered	Adults diabetes patients	Patients with diabetes	- Disinterest in portal use was linked to patient satisfaction with the patient–provider relationship, so as participants with a satisfying provider–patient relationship appeared less in need of the patient portal. - Barriers to learning the system such as lower computer literacy and the time required to learn - Fear of losing personal communication with their primary physicians through emails (outside of the portal functionality) - Concern about who in the office would be reading the e-mail messages sent over the portal because of the indirect routing of the e-mails sent through portal
Britto et al. (2009) [38]	To evaluated the usability of “MyCare Connection” portal for parents of children with cystic fibrosis, diabetes or arthritis.	Mixed	The USA	Web-based secured web application allowing to view demographic and contact information; laboratory, radiology and pathology reports; inpatient and outpatient encounters; medications; and secure electronic messaging	Tethered	Children with cystic fibrosis, diabetes or arthritis	Parents of children with cystic fibrosis, diabetes or arthritis	- Clarity of information and the ability to comprehend error messages was scored least in the satisfaction study - A number of problematic usability issues including: use of medical jargons and terminology; problematic clarity of normal and abnormal values; information overload and information complexity requiring medical interpretations and explanations; more help options and bolder and more eye-catching sidebars and instructions were needed
Kim et al. (2009) [51]	To assess the use and utility of PHRs in a low-income, elderly population	Quantitative	The USA	A stand alone, individually controlled, Web-based repository of personal health information allowing users to enter, update, or delete structured information in nine different categories. It provides summary pages that list all the information entered into the system by the user. A hardcopy and/or electronic copy can be shared with health care providers or family members.	Untethered	Adults elderly chronic patients	Elderly residents of a nursing home with chronic diseases	- Most (77%) of the system use happened while assistance from graduate nursing students or housing staff was available to the residents.
Sarkar et al. (2010) [69]	To examine use of an internet-based patient portal among a well characterized cohort of English-speaking adult patients with diabetes differed between those who report limited health literacy versus those who do not.	Quantitative	The USA	An internet based patient portal allows viewing laboratory test results, sending email to providers, requesting medication refills, and making medical appointments.	Tethered	Adults diabetes patients	Diabetes patients	- African-America, Latino, and Filipino race/ethnicities and lower educational attainments were associated with increased risk of not signing on to the patient portal. -Those with limited health literacy had higher odds of never signing on to the patient portal
Weppner et al. (2010) [84]	To Evaluate use of a web-based shared medical record (SMR) between older patients with diabetes and providers.	Quantitative	The USA	A web-based shared medical record allowing a secure messaging with health care providers, request medication re-fills and in-person appointments; and view test results, after-visit summaries, medical problem lists, allergies, and immunizations.	Tethered	Adults diabetes patients	Diabetes patients	- Unadjusted analyses indicated that younger age, male sex, living in a higher socio-economic neighborhood, and primary care physician level of secure messaging were associated with patients’ initial portal use - Higher morbidity of patients was linked to higher signing up and continued use of the system
Wagner et al. (2010) [83]	To examine patient perspectives on ePHR use and functionality as part of the development process of an existing ePHR	Qualitative	The USA	My HealthLink, an ePHR enabled consumers to store personal health information with core functions of secure messaging; access to educational materials; medication interaction checking; recording and monitoring health measures, for example, blood pressure; and goal setting and health diaries.	Untethered	Adult patients with hypertension	16 patients with hypertension	- User themes requiring attention: some difficult to understand terminology, changing relationship with providers, overwhelming and time consuming task of using PHRs, cost of ePHR - Technology themes mainly reflected on health and technology literacy, patient usability, ease of access, need for additional instructions, and the potential of customizable menus. - Linkage to the other systems - Desire to choose those providers which have access to patients’ ePHR
Nordfeldt et al. (2010) [59]	To explore patients’ and parents’ attitudes toward a local Web 2.0 portal tailored to young patients with type 1 diabetes and their parents and opportunities and obstacles to the application of the system	Qualitative	Sweden	A Patient portal called “Diabit” containing specific diabetes-related information and social networking functions such as message boards and blogs and allowing medical prescription renewal, making appointments, sending questions, viewing questions and answers, contact information, photos of staff, and other general information about the local diabetes teams and their services. Used by patients, parents and providers.	Not documented	Children with diabetes	16 mothers and 3 fathers of sick children, and 5 young patients (11-18 years old)	- The experience of already being in control and having felt secure with the treatment over a long period of time was one reason for limited use of the portal. - Previous good contact with the practitioners, good continuity over time regarding such relationships, sufficient personal experience with living with diabetes, and perceived long-term success regarding treatment were mentioned as factors that might contribute to a lower perceived need for repetitive use of the portal - Various unsuccessful user experiences, such as few hits from a specific search or seeing that there had been little activity in the practitioners’ news and updates sections of the portal, could create the perception that the practitioners were not “on their toes” - Issues with passwords - Users with particularly negative feelings about their disease and/or health care experiences might not be willing to go through the procedure for logging onto a disease-specific portal.
Goel et al. (2011) [47]	To identify patient reported barriers to enrollment in a patient portal among patients who did not enroll despite being directly offered this service by their providers	Quantitative	The USA	MyChart, a patient portal allowing a patient to log-on to a secure portal to access personalized health information, including laboratory results and a medication list and sending secure electronic messages to physicians.	Tethered	Adult chronic patients	Chronic patients including diabetes, hypertension, chronic pulmonary disease, coronary artery disease, congestive heart failure, peripheral vascular disease, severe chronic liver disease, renal failure, cancer, and dementia	- Reasons for not enrolling: did not remember discussing the patient portal with their providers (26%), did not attempt enrollment despite remembering a discussion with their providers (63%), and attempted to enroll but did not succeed (11%). - Reasons for not attempting to enrollment: 60% stated reasons related to lack of information or motivation, 30% reported negative attitudes toward the patient portal, and 8% reported connectivity obstacles - There were large, but non-significant differences in reasons for not attempting enrollment by race; black people mentioned more negative attitudes and connectivity obstacles - There were large differences in reasons for not attempting enrollment by presence of chronic disease (lack of information/motivation was cited by 55% with chronic disease vs. 71% without chronic disease) - Additional reasons for not attempting enrollment: 37% said they prefer to call the providers’ office to discuss health matters rather than communicate electronically and nearly 25% reported they did not feel the internet is a safe way to communicate sensitive health information.
Tenforde et al. (2011) [4]	To measure the association between use of an advanced electronic medical record-linked PHR and diabetes quality measures in adults with diabetes mellitus (DM).	Quantitative	The USA	MyChart, the Cleveland Clinic’s electronic medical record (EMR)-linked PHR, allowing to access patient’s’ diagnoses and co-morbidities, laboratory and other test results, along with secure messaging through the PHR with their provider. Patients can also access glucometer readings, a set of diabetes-related health and wellness links, and diabetes specific health reminders (including recommended glycated hemoglobin, urine albumin, and cholesterol testing due dates, recommendation for pneumococcal vaccination, and due dates for diabetic foot and dilated retinal eye exams).	Tethered	Adult diabetes patients	4,036 diabetes patients	- Compared to non-users, PHR users were younger, had higher incomes and educational attainment, were more likely to be identified as Caucasian, and had better unadjusted and adjusted diabetes quality measure profiles
Sarkar et al. (2011) [70]	To examine patient use patterns of an innovative internet-based patient portal within a well-characterized large, diverse cohort of adult medically insured patients with diabetes	Quantitative	The USA	An internet-based patient portal allowing to view laboratory test results, email physicians or care team, request medication refills, and make appointments.	Tethered	Adult diabetes patients	5671 diabetes patients	-African-American (31%), Latino (34%), and Filipino (32%) participants least likely, and Asian (53%) and White (51%) participants most likely to both request a password for the internet-based patient portal (a marker for internet access and intent to use) and log on to the portal after requesting a password - Compared to non-Hispanic Caucasians, African-Americans and Latinos had higher odds of never logging on, as did those without an educational degree compared to college graduates - Age over 70 years was associated with lack of use among the entire cohort - Compared to those who used the patient portal, nonusers were more likely to have suboptimal control of their diabetes and related risk factors
Nielsen et al. (2012) [57]	To evaluate the use of a secure internet portal in an academic Multiple Sclerosis (MS) Center	Quantitative	The USA	“PatientSite”, a patient internet portal allowing individuals to manage their clinic appointments (making, canceling, or rescheduling with department administrators), request prescription refills and referrals directly to their physician’s office, view their medical records including labs, pathology, and radiology study results, and communicate directly with their provider regarding non-urgent issues through a secure electronic message system. In addition, PatientSite provided web links to helpful health-related information, an account statement for patient medical bills, and technological support to portal users	Tethered	Adult multiple sclerosis patients	240 multiple sclerosis patients	- Portal users tended to be young patients with minimal physical disability. Independent predictors and barriers of portal use include the number of medications prescribed (OR 1.69, *p*<0.0001), Caucasian ethnicity (OR 5.04, *p*<0.007), arm and hand disability (OR 0.23, *p*<0.01), and impaired vision (OR 0.31, *p*<0.01). - Barriers to portal use included being a minority (0.2-fold odds), worse visual acuity (0.31-fold odds) and upper extremity function (0.23-fold odds). - The number of clinic visits scheduled was greater among portal users compared to non-users (*p*<0.0001). A trend toward a greater proportion of ‘no-shows’ to clinic was found among portal non-users (4.2%±10.7 vs. 2.1%±7.3, *p*=0.12).
Wagner et al. (2012) [82]	To examine the impact of a PHR in patients with hypertension measured by changes in biological outcomes, patient empowerment, patient perception of quality of care, and use of medical services.	Quantitative	The USA	My HealthLink, which provided a secure, comprehensive, electronic record that enables consumers to store PHI. This PHR is “ allowing to view problem lists and information on medications, allergies, and immunizations Core functions also include: secure messaging; access to educational materials; medication interaction checking; recording and monitoring of health measures, for example, BP; and some goal setting and health diaries.	Tethered	Adult patients with hypertension	443 hypertensive patients	- Younger age, self-reported computer skills, and more positive provider communication ratings were associated with frequency of PHR use vs. no use. - In multivariate analysis, patients from Family Medicine (versus those from Internal Medicine), those with a greater number of self-reported internet-use items, and higher provider communication scores had significantly more frequent PHR use
Day and Gu (2012) [41]	To find out: what factors influence PHR use? Do perception of ease of use influence patient’s engagement with the software? What is about available software that is considered useful by patients?	Qualitative	New Zealand	PHR linked to their doctor's Practice Management System (PMS) allows viewing laboratory results, diagnosis, immunizations and medications list Capabilities: interaction patients with their GP, singing patients to system via internet at home and accept electronic invitations	Tethered	Adults chronic patients	Chronic patients (not specified)	- Required computer and health literacy which contribute to being able to effectively use the PHR - Usability issues (e.g., navigation in general was not intuitive and some PHR functions were not useful) - Concerns about how PHR-related services are paid for, who pays and under what circumstances and necessity for incentive motivation (e.g., getting a fixed number of free consultations and paying for extra)
Emani et al. (2012) [42]	To apply a theoretical model, the diffusion of innovation model, to the study of PHRs and conduct an exploratory empirical study on the applicability of the model to the study of perceptions of PHRs	Quantitative	The USA	Patient Gateway, allowing requests for appointments, prescription refills and referrals, access to certain components of the EHR such as laboratory results, and secure messaging with the practice and provider	Tethered	Adult asthma, CHF, hypertension, or diabetes patients	Asthma, CHF, hypertension, or diabetes	- Computer use among non-adopters (75%) was lower than that among PHR users (99%) and rejecters (92%) (*P* < 0.001). Non-adopters also reported a lower score on personal innovativeness in information technology. - Innovators were younger than other users and non-adopters (*P* = 0.001) - Only 50% of non-adopters had a four-year college degree or more compared to 76% of the innovators, 71% of laggards, and 69% of other users (*P* = 0.001). - Only 41% of non-adopters had a total household income of $75,000 or more compared to 75% of laggards, 72% of innovators, and 63% of other users (*P* < 0.001). - Non-adopters also differed from innovators and laggards on marital status (47% married; *P* < 0.001). - In terms of overall health status, non-adopters reported a lower rating of overall health compared to innovators and laggards, and other users and rejecters reported lower overall health status than innovators. Innovators also reported a smaller number of comorbidities (mean = 2.8) than other users, rejecters, and non-adopters (mean = 3.7). -The greater the relative advantage, ease of use, and trialability of the PHR, the more patients value the PHR for communicating with their doctor’s office. - More positive perceptions of privacy and security of information in the PHR are associated with greater perceived value of the PHR.
Tom et al. (2012) [78]	To examine integrated personal health record use patterns among parents of children with chronic disease and compare ratings of care experiences between integrated PHR users and nonusers.	Quantitative	The USA	In “MyGroupHealth” parents access their child’s account as a proxy through their own account. Users can viewing: immunizations, test results, after-visit summaries, allergies, medical conditions, health assessments, health plan benefits and medication management Capabilities: secured messaging and appointment management.	Tethered	Children with chronic disease	Parents of a child with at least one chronic disease (types not specified)	- The top reasons for not using the PHR among nonusers were “too busy”, “forgot login name and/or password”, and “child does not have health care needs” - Some participants noted that they were not comfortable sharing medical information on the Internet - Other reasons to not using the PHR: forgot login name and/or password; too difficult to get online access for the PHR; not having access to the high-speed Internet; too difficult to use; no response from system; not sure how to use the Internet - Preference of other routes of care (e.g. face to face) instead of the PHR
Urowitz et al. (2012) [79]	To evaluate the experience of patients and providers using an online diabetes management portal for patients.	Qualitative	Canada	A Patient portal which provides access to “Health Library” for diabetes education material (for both patient and providers) and providers access to “Personal Health Records” for allowing patients to consolidate their personal health information including medical and family history, medication details, lifestyle choices, and test results	Tethered	Adults patients with diabetes	Patients with diabetes and their providers i.e., general practitioners (GPs), nurses, nurse practitioners (NPs), dieticians, diabetes educators (DECs), and other clinical staff	- Technical issues regarding usability and discoverability (e.g. access to the internet, difficult data entry, and difficulty in finding items) - Some patients felt that they were controlling their diabetes well or found that their health measurements had been fairly stable and therefore did not feel the need to enter information. - Required provider duplicate time and efforts to handle issues related to the PHR use parallel to those in the office time, then viewed it as a tool for patients and other care providers - Provider concern on overreliance of patients on portals when exacerbations in their condition occur
Gordon et al. (2012) [87]	To describe the process and outcome of developing and implementing a personal health record for people living with HIV/AIDS	Mixed	The USA	My health profile allowing to access most recent medication lists, test results, information on healthcare providers and payers, viewing an integrated audit log, and enabling the development a continuity of care document	Tethered	Adult	Patients living with HIV	- Potential barriers to use of My Health Profile including functional and computer literacy, privacy and confidentiality concerns, potential reluctance to use technology, and cognitive challenges (e.g., remembering passwords) - PHR implementation was well matched with the organizational mission and values and priorities related to coordination of care
Logue et al. (2012) [54]	To describe the results of an exploratory study that provided an initial test of a theoretical framework to understand an elderly’s decision to participate in self-directed care	Quantitative	The USA	Without a PHR	Not applicable	Adult chronic condition	Senior adults with chronic conditions	- Older seniors reported less confidence in their ability to use internet-based PHRs and did not perceive that they had the resources in place to use them. - More men than women agreed that they had access to care, access to the internet, enjoyed computers, saw PHRs to be a better fit with their healthcare needs, and expressed confidence in using the internet to communicate with others and in using an internet-based PHR. -ethnicity - Older seniors were less likely to know how to find health resources on the internet and were less interested in observing the use of PHRs. - Those who knew more about what health resources were available on the internet were more likely to be motivated by incentives to use PHRs. - Older seniors were less confident in their ability to self-manage their own health. By contrast, older adults did not report less computer access; however, they did have less access to and familiarity with the internet - Easier access to care was positively correlated with believing that PHRs offer an advantage over alternative methods, that PHRs were compatible with their current healthcare needs and that PHRs were likely to give them the results that they expected. Those that reported easier access to care also were more likely to express confidence in their abilities to communicate via written language and self-manage their health - Of the respondents who disagreed or were undecided (relating to the three e-health indicators), 51% (n = 18) reported not having access to a computer and 49% (n = 17) reported not having access to the internet. These results indicate that internet access is a prerequisite to knowing what, where and how to find health resources via the internet - More females (64%) than males (20%) reported not knowing how to use internet-based PHRs; - The intention to use PHRs within the next year was positively correlated with the likelihood of accepting incentives to use them. In addition, incentive motivation was positively correlated with an individual’s confidence in using an internet-based PHR and the likelihood that they would choose a provider who uses it - Many more females (28%) were worried about privacy compared with males (10%) - Those who preferred to work together with their healthcare provider as a team were more likely to be motivated to learn new things, know what health resources were available via the internet, believe that using an internet-based PHR would give them the health outcomes they sought, be incentivised to use PHRs, prefer to control who could access their PHR see a fit between their current healthcare needs and PHRs, be interested in trying one - Positive correlations were also noted between the number of illnesses the person reported and PHRs fitting their current healthcare needs. Respondents with more illnesses were more likely to choose a healthcare provider based on the provider’s use of information from their PHR. Those with multiple healthcare providers were the same people who preferred to manage their own health, intended to use a PHR within the next year, believed that PHRs were compatible with their current healthcare needs and would choose a provider based on the provider’s use of the information from their PHRs
Britto et al. (2013) [37]	To examine parents’ perceptions of the benefits and / or drawbacks of a patient portal for managing their child's chronic illness.	Qualitative	The USA	A secure Internet-based application which integrated to an EHR Users can viewing: laboratory results, visit history, medication information Capabilities: secure messaging to health care providers, upload documents and share with health care providers and reminders for laboratory tests and clinic visits.	Tethered	Children with Cystic fibrosis, Diabetes mellitus or Juvenile idiopathic arthritis	Parents of children with cystic fibrosis, diabetes mellitus or juvenile idiopathic arthritis	- A potential concern on the loss of interpersonal contact with providers and some parents’ preference for direct communication, particularly when hearing bad medical news - A concern about not knowing who would receive electronic communications and whether anyone would answer
Osborn et al. (2013) [61]	To (1) understand who uses an existing patient portal and reasons for use and nonuse, (2) understand how portal users are using a portal to manage their medications, and (3) explore participants’ ideas for improving portal functionality for medication management and adherence support.	Mixed	The USA	MyHealthAtVanderbilt, a patient portal allowed managing medical bills, viewing PHI (eg, vital signs, laboratory results, medication lists, and diagnoses) from their electronic health record (EHR), using secure messaging to communicate with providers and manage medical appointments, and view educational contents	Tethered	Adults diabetes patients type 2	75 adults with type 2 diabetes	- Users were more likely than nonusers to be Caucasian/white, have higher incomes, and be privately insured. Users also tended to have more education than nonusers - Reasons for nonuse included not knowing about the portal, not having access to a computer, or having a family member serve as an online delegate.
Ronda et al. (2013) [67]	To study the characteristics, the health status, the self-efficacy, the diabetes knowledge, and the treatment satisfaction of patients with diabetes who do and do not have a login for a patient Web portal	Quantitative	The Netherlands	A patient portal allowing users to access their medical records, including the information provided by their healthcare provider during medical consultation, such as physical examination, laboratory results, problem lists, and treatment goals. It also provides access to general diabetes information and an overview of all examinations and diabetes visits that are needed according to guidelines. Patients can upload the glucose levels measured at home and seek contact with their care provider through secured electronic messaging	Tethered	Adults diabetic patients types 1 and 2	Diabetic patients of 18–85 years old	- The participants with a login were significantly younger compared with those without. Of the participants with a login, 63.1% were male compared with 56.5% of the group without login. - In Type 1 diabetes: patients with a login were younger and had a higher education level. Following the guidelines, most type 1 diabetes patients were treated by an internist; however, patients without a login were more frequently found to be treated in a general practice. Type 2 diabetes: patients with a login had been diagnosed with diabetes for a longer time, and used insulin more frequently and also used more other drugs compared to those without a login. - Patients without a login significantly perceived less diabetes-related distress than patients with login and also had less self-efficacy and lower diabetes knowledge. - With increasing age, the odds of requesting a login decreased. Also, the odds of requesting a login increased in males, in patients with a higher education level, in patients who speak Dutch fluently, and in patients with a paid job, whereas the odds decreased in patients treated by a primary care physician (vs. an internist) or living alone.
Wade-Vuturo et al. (2013) [81]	To identify the benefits of and barriers to using secure messaging (SM) within a portal.	Mixed	The USA	A patient portal which called “MyHealthAtVanderbilt (MHAV)”. Users can view EHR data, use secure messaging to communicate with providers, manage medical appointments and bills, and perform other tasks.	Tethered	Adult diabetes patients type 2	Patients with type 2 diabetes	- Barriers to using SM: (a) preconceived beliefs about technology or rules about SM (e.g., the questionable reliability of the patient portal to facilitate a timely and productive message exchange with their providers, (b) prior negative experiences with SM (e.g., not receiving a response to a patient-initiated message). - Perceptions of provider endorsement of SM i.e., (a) participants’ assumptions about providers’ willingness to use SM, providers being interrupted by SM, and providers not being reimbursed for SM, (b) providers’ instructions about SM (e.g., participants recounted instances when providers instructed them not to use SM)
Lyles et al. (2013) [55]	To examine the associations between patient ratings of provider communication or trust with portal use in diabetes patients.	Quantitative	The USA	Patient portal allowing users to view visit summaries, medical history, and/or immunizations/allergies, making appointments, order medication refills, view the results of medical tests, and send or receive secure electronic messages with providers.	Tethered	Adults diabetics patients	Diabetes patients	- There were a significant but modest adjusted association between increased trust and being a registered user - Among patients ≥70 years of age, there was a significant association between patient-provider communication and portal use -There were also significant association between trust in provider and race/ethnicity and age. Latino subjects were more likely to be a registered user when there was higher trust in the provider, as were white patients and patients ≥70 of age - After adjustment, there was a significant association between trust in provider and overall secure message use
Pai et al. (2013) [62]	To determine the experience of, and feedback from, prostate cancer patients using a PHR, while receiving care from a provincial cancer agency.	Mixed	Canada	“Provider”: a web-based integrated with an electronic clinical information system to store and access the medical records of patients with cancer. With access to laboratory, pathology, imaging, operative, and procedure reports, scheduling and appointment information and medications, secure messaging patient-provider and etc.	Tethered	Adult patients with prostate cancer	Male patients with prostate cancer	- Mixed responses and lack of clarity on who should pay for the PHR, for example, federal government, provincial government, cancer agency (that is, health care providers), donations or charities, private industry, clients (that is, patients), and other. Besides choosing other options, patients felt that the government should help fund the PHR. - Several operational difficulties with the “Provider” Web site were reported by both patients or the research assistant
Martinez et al. (2013) [89]	To identify the characteristics of PHR users versus non-users	Quantitative	Argentina	A web-based PHR allowing patients to view laboratory results, diagnosis, preventive information and medications lists and also to communicate with doctors or health care systems (e.g., for massaging system, appointments scheduling or medication delivery, and to get support for self-management)	Tethered	Adult chronic patients	Chronic patients with hypertension, diabetes, dyslipidemia, cerebral vascular disease, coronary artery disease, chronic heart failure, chronic renal failure, peripheral vascular disease, and smokers	- PHR users were younger and women and had at least one disability or chronic condition and had asked for medical assistance during the last year - The main predictor of PHR use was being a patient asking for medical assistance during the last year, increasing the PHR use by almost 4 times.
Luque et al. (2013) [88]	To assess barriers and facilitators to use of online PHRs among persons living with human immunodeficiency virus	Mixed	The USA	Using an exemplary PHR	Tethered	Adult	Patients living with human immunodeficiency virus	- Lack of computer or broadband access and also privacy when accessing a portal outside of one’s home were mentioned as important barriers; computer literacy as a barrier but not as an insurmountable one - Barriers to the use of the Internet cited by respondents were cost (16/90,18%), lack of interest (6/90, 22%) and do not know how to use (5/90, 19%).
Byczkowski et al. (2014) [39]	To assess parents understanding of the importance of PHR, their concerns for using web-based portals for their children’s diseases	Mixed	The USA	A web based patient portal allowing access laboratory result, medication information, and a child's visit history	Tethered	Children with cystic fibrosis, diabetes mellitus, and juvenile idiopathic arthritis	530 parents of children with cystic fibrosis, diabetes mellitus, and juvenile idiopathic arthritis	- 12 percent mentioned that they sometimes saw information in the portal that frightened them, and 11 percent reported that they sometimes see information that they would have preferred to get directly from their provider. - Requests by parents for easier access to the system and navigation through it, more personalized information according to the child's condition, more medical terminology explanations, and notifications for new lab results
Fiks et al. (2014) [44]	To design a portal to facilitate shared decision making between families of children with asthma and primary care clinicians based on user-identified criteria and integrated within the EMR	Qualitative	The USA	A Patient portal which called “MyAsthma” and it’s was designed to work within the framework of an existing patient portal, MyChart, and was linked to The children’s Hospital of Philadelphia’s EMR through a Web-based framework	Tethered	Children with asthma	7 parents of children with asthma and 51 care providers including pediatricians, nurses, and a pharmacist	- Preference for direct communication with physicians - System interface should be simple and the content be clear - Provider concern: should be viewed as access to care for chronic condition and not for an acute flare in the condition
Sharp et al. (2014) [72]	To characterize the knowledge, interest, and attitudes of childhood cancer survivors and their caregivers towards ePHRs.	Qualitative	The USA	Without a specific PHR	Not applicable	Children with cancers	Caregivers of survivors who were <14 years old and also survivors ≥14 years old along with their caregivers when present	- Data security and privacy were the primary concerns expressed by those who had a concern. However, among them, 67% of survivors and 80% of caregivers stated that the concern would not prevent them from using an ePHR.
Odlum et al. (2014) [60]	To assess the ease of use and usefulness of My Health Profile (MHP) and to identify the actual information needs of MHP-users and perceived information needs of MHP-users and MHP non-users before MHP-plus roll out.	Mixed	The USA	MyHealthProfile, a continuity of care document enabling access to facets of medical records through the internet	Untethered	Adults people living with HIV	People living with HIV	- Problematic issues including incomplete health information in the MHP (e.g., lacking vaccinations and diagnostic test results); and confusing information display in MHP. - Participants expressed the need for health information to better facilitate provider visits. - Frustration about how to grant providers access, and whether they know how to use the system
Barron et al. (2014) [35]	To explore whether older adults with chronic conditions and/or their caregivers demonstrate capacity to use a patient portal, and their perspectives on the experience	Qualitative	The USA	A patient portal enables accessing the P/A/M/I lists, office notes, hospital discharge summaries, and test results	Tethered	Adult chronic obstructive pulmonary disease or congestive heart failure	14 patients and 19 caregivers	- Usability issues related to unfamiliar medical terms, font and color contrast issues, and poor section labeling
Baudendistel et al. (2015) [36]	To explore needs and requirements of potential users with regard to the content and function of a patient-controlled personal electronic health record	Qualitative	Germany	Without a PHR	Not applicable	Adult patients with colorectal cancer	Patients with colorectal cancer, health care providers, clinicians, clinical staff in an umbrella company	- Needs and requirements: a structure necessary to facilitate tracking the course of illness and treatment over time for both physicians and patients; highlight important or new information with color or a priority for current issues; include a basic dataset of relevant information that would be crucial for everyone involved in the patients’ health care with manageable volume of information - The presentation of information should be in a patient assessable, accessible, and comprehensible way. - Given the fact that several physicians from different health care settings would have PHR access, physicians expressed concerns and uncertainty regarding negative consequences on professionals’ liability for reacting to patient-added information or commentaries
Gartrell et al. (2015) [45]	To examine factors associated with ePHR use by nurses for their own health management	Quantitative	The USA	Different PHRs	Not applicable	Adults chronic patients	664 sick nurses with chronic conditions in 12 hospitals	- A larger percent of ePHR users had a chronic medical condition and/or were taking a prescribed medication (71%) compared to non-users (65%) (*P*<0.05) - A large portion of PHR users used the Internet frequently (several time per day) compared with PHR non-users (*P*<0.05) -A larger portion of ePHR users were more aware of health technologies; and nearly 80% of users compared with approximately 50% of nonusers indicated their primary care providers currently used an EHR for care (*p*<0.01) - More ePHR nonusers (72%) were concerned about general privacy and security of health information online compared with users (64%, *p*=0.06) - Factors associated with ePHR use: being an active healthcare consumer (having a chronic health condition and taking prescribed medications) and having a healthcare provider using a EHR for care,
Gee et al. (2015) [46]	To learn from chronically ill engaged and educated (e-patient) adults how and why they use PHRs for self-management support and productive patient-provider interactions.	Qualitative	The USA	Different PHRs (Core functions were not documented)	All tethered	Adults chronic patients	18 chronic patients	- Health and computer literacy issues (e.g., understanding numbers related to their individual health conditions and navigating through the PHR) - Usability issues (e.g., the use of medical jargons) - Frustration following seeing incomplete or incorrect profile, medication, and medical history data in the PHR but not being able to intervene through the system. - Lack of proper or adequate user training on the use of PHR - Concerns about privacy of personal or banking data and information - Provider related issues (e.g., request for involving other providers such as pharmacists, therapists, dietitians); lack of clarity about who may see the content of their messages (i.e., their physicians or office staff); providers not using the PHR; and concerns about the provider workload following PHR use - A lack of interoperability between systems in provider offices and other systems and the resulting frustration related to care coordination between offices that use EHR/PHR systems and those still using paper-based systems.
Harrison et al. (2015) [10]	To understand perceptions of CKD patients about ePHRs, and describe characteristics associated with their expressed intent to use an ePHR.	Quantitative	Canada	Without a PHR	Not applicable	Adults patients with non-dialysis-dependent CKD	Patients with non-dialysis-dependent CKD	- Patients over the age of 65 were less likely to intend to use an ePHR - No association between gender or self-perceived health and intent to use the ePHR - Those with post-secondary education and Internet access were more likely to express their intent to use an ePHR - 69.8 % of our patient group intended to use an ePHR if it became available - Patients who did not convey intent to use the ePHR did not report anticipated benefit of ePHR use as often -The perceived benefits of greater personal involvement in healthcare, access to health information and lab results were associated with expressed intent to use - Privacy of health records as the most common concern noted regarding ePHR use
Nippak et al. (2015) [58]	To explore the perceptions of family members regarding the importance of an electronic personal health record to support the care of their loved ones within a long term care facility	Mixed	Canada	MyChart: a secure and private web-based platform that offers self-management tools that are entirely accessed and controlled by patients such as diaries to record their health history, symptoms, and medications, emergency contact information as well it provides access to health education sites and appointment scheduling features	Not documented	Adults elderly chronic patients	Family members of residents residing in a long term care facility	Family members identified concerns linked to: - Privacy, confidentiality, and security of the electronic health record information - The knowledge and understanding e.g., not understanding the information and fearing abnormal values - Issues related to the use of MyChart on staff operations and their communication exchange with family members such as staff workload and impact on communication between staff and patients
Tieu et al. (2015) [76]	To explore the barriers and facilitators to use of a patient portal in anticipation of portal implementation in an urban, safety net primary care clinic	Qualitative	The USA	Patient portals in general/without a specific PHR	Not applicable	Adult chronic patients	11 patients with chronic illness including diabetes and 5 caregivers	- Health and computer literacy challenges (e.g., problems with reading and typing; personal experience with online security breaches/viruses, and distrust of potential security measures; lack of basic computer skills and problems of handling passwords; challenges with the medical terminology and lack of language-appropriate information) - Concerns about the affordability of the Internet, particularly the cost of mobile data - Concern about privacy, confidentially and security of patients’ health information particularly sensitive diagnoses and medications being online or easily accessible to researchers and industry members - Concern over technology replacing the healthcare providers and diminishing or interfering with ongoing in-person communication with them
Wells et al. (2015) [12]	To investigate organizational strategies to promote PHR adoption with a focus on patients with chronic disease.	Mixed	The USA	Different PHRs across the country	Not applicable	Adult chronic patients in general	Chief information officers, directors of e-health services, medical directors, or internists with specialized roles in chronic disease management, quality, primary care, or population health	- The greatest barrier to PHR implementation was perceived to be physician resistance due to concerns about the impact on their workload and on their patients. - Discussions around how to better reimburse provider interactions by PHR
Latulipe et al. (2015) [52]	To investigating facilitators and barriers to adoption of patient portals among low-income, older adults in rural and urban populations	Qualitative	The USA	A patient portal in general/Without a specific patient portal	Not applicable	Adults chronic patients	36 chronic patients and 16 caregivers (chosen from low-income, older adult populations across the country)	- Lack of interest in technology in general and portal use (15 out of 36 patient participants) as well, which appeared to be linked to age - Lacking confidence with respect to technology use especially in older participants (e.g., remembering passwords) - The most frequent concern noted was that of privacy and security (e.g., leading to for example misuse of information by insurance companies to deny coverage) - A fear that use of the patient portal could eventually replace face-to-face visits with their healthcare provider and this was seen as a potential negative consequence of signing up - Stress following reading medical information instead of hearing them - Unclear from who to get technical assistance in the case of a problem
Smith et al. (2015) [74]	To document disparities in registration and use of an online patient portal among older adults.	Quantitative	The USA	A patient portal allowing three main options (message a provider, request a prescription reauthorization, and view test results) with additional options including personal health records (monitoring vital statistics [e.g. height, weight, body mass index, body surface area, blood pressure, heart rate, breathing rate, temperature], previous conditions, and current conditions), previous or upcoming appointments, sent and received messages, personal profile, and a help page.	Tethered	Adults with chronic conditions including arthritis, asthma, bronchitis or emphysema, cancer, coronary heart disease, depression, diabetes, heart failure, and hypertension.	534 older adults with chronic conditions including arthritis, asthma, bronchitis or emphysema, cancer, coronary heart disease, depression, diabetes, heart failure, and hypertension.	- White patients, male gender, college graduates and those with marginal or adequate health literacy were more likely to have registered their patient portal and use its options.
Eschler et al. (2016) [43]	How do individuals characterize their experiences of and expectations for using asynchronous communication strategies to coordinate health care with clinicians?	Qualitative	The USA	A patient portal allowing to view medical test results, visit summaries, immunization lists, allergy lists, medical condition lists, exchanging secure messaging with providers, ordering medication refills, scheduling an in-person appointment	Tethered	Children with asthma and adults with diabetes	7 parents of children with asthma and 12 adult diabetes patients	- Failing to track issues following a secure communication with care providers such as a lack of status indicators for unresolved issue; and exposing patients to inconsistent communication patterns such a by either a call or a written note, which confuses patients leading to potential lapses in illness management
Schneider et al. (2016) [71]	To understand patients’ lived experience with a patient-controlled electronic health record (PCEHR) and how the use of such a technology may lead to patient empowerment	Qualitative	The UK	A patient-controlled electronic health record, called Patients Know Best It allowed patients and clinicians alike to upload, enter, view, and edit various health data (e.g., symptoms, medications, diagnoses, test results, and body measurements). It also provided features such as electronic messaging, video conferencing, and file management.	Untethered	Children with chronic gastrointestinal diseases	16 parents of sick children and a teenager who was ill as well as 11 clinicians	- Patients’ willingness to take power and responsibility for their health through using technology depends heavily on the patient’s coping style and perceived competence, autonomy, and relatedness. - Failure of the system in its chronic care context to meet the needs of chronic patients and their caregivers who followed the avoidance-oriented coping style towards their chronic condition, which typically involved denial and suppression of their condition and disengagement in chronic care. - The avoidance-oriented people used the system only when necessary to coordinate care or to communicate with the clinical team, or did not use it at all, when compared to the approach-oriented people who were found to use the system heavily to track symptoms, medication, and food intake and to investigate test results.
Hazara and Bhandari (2016) [49]	To evaluate the characteristics and experiences of those patients who have registered for renal patient view but were inactive in using it.	Mixed	The UK	A renal patient view that is a secure website to view and monitor lab results, to document and monitor certain health parameters that of interests for their renal care providers e.g., weight, blood pressure, blood glucose and medications. It also includes educational materials	Tethered	Adult renal chronic patients	69 chronic renal patients	- Main reasons for being in-active were mentioned as difficulties in using computers or passwords (45%) followed by the perception that it did not add anything to the members’ existing relationship with their renal team (37%) - Other reasons: being too busy, getting anxious when seeing results online, difficult website navigation - No need to use the website due to satisfaction with the routine communication with the renal care team
Graetz et al. (2016) [48]	To understand whether socio-demographic differences in patient portal use for secure messaging can be explained by differences in internet access and care preferences.	Quantitative	The USA	A patient portal allowing users to view lab test results, order prescription medication refills, schedule nonurgent primary care visits, view after-visit summaries, and exchange secure electronic messages with their health care providers.	Tethered	Adult patients with asthma, coronary artery disease, congestive heart	1041 patients aged 18 or older who had at least one of the following chronic conditions of asthma, coronary artery disease, congestive heart failure, diabetes, or hypertension	- Without adjustment for internet access or care preference, patients who were male, older, of Asian or black race/ethnicity, lower income, and with less education were statistically significantly less likely to have used the portal to send a secure message than those who were female, younger, of white race/ethnicity, higher income, and higher education (*P*<0.05). - Frequency of internet access was associated with higher use of the portal - Patients who reported a preference for getting care in-person or over the phone instead of online were less likely to report having used the portal to send a secure message (*P*<0.001)
Ryan et al. (2016) [68]	To explore the feelings, ideas and expectations of patients and primary care providers concerning both the implementation and the use of patient portals.	Qualitative	Canada	Without a patient portal/patient portal in general	Not applicable	Adult patients with diabetes, hypertension, asthma, obesity, COPD, thyroid condition, hyperlipidemia and cancer.	7 patients and 4 providers (i.e., two family physicians, one nurse practitioner and one family practice nurse)	- Challenging or problematic issues related to the accessibility of patient portals regarding computer literacy and the cost of portals (especially if patients need to pay), impact on provider workload, the primacy of direct patient-provider relationship, honesty and trust on the data entered by patients, privacy and confidentiality of information, ability of patients to understand and interpret the content of portals
Arcury et al. (2017) [34]	To determine potentially modifiable factors affecting patient portal utilization by older adults who receive care at clinics that serve low income and ethnically diverse communities.	Quantitative	The USA	The patient portal systems of the urban and rural clinics differed; but included viewing test results, sending a message to doctors or nurses, refilling prescriptions, making or changing an appointment, requesting a referral, finding information about a health issue, and other	Tethered	Adult patients with diabetes, hypertension, dyslipidemia, or cardiovascular disease	100 patients with diabetes, hypertension, dyslipidemia, or cardiovascular disease	- Patient portal utilization did not differ by participant age or gender - Poverty level was associated with patient portal utilization: 91% of those below the poverty level, 74.4% of those at 100% to 200 % of the poverty level, and 53.1% of those above 200% of the poverty level had not utilized their patient portal - Those with greater than a high school education had greater odds of patient portal utilization . Those who were not currently married had lesser odds of patient portal utilization - Receiving care at an urban clinic greatly increased the odds of patient portal utilization -Those with worse health utilized their patient portal more - More minority participants (90.8%) than white participants (62.5%) had not utilized their patient portal - Lesser eHealth literacy was associated with patient not utilizing their portal -Those with access to e-devices and Internet in their homes (33.9% vs 1.2%), who use the Internet at least once a day (47.5% vs 8.6%), and who experience no stress when using a computer (50.0% vs 11.3% who experience at least some stress) were more likely to utilize their patient portal.
Sieck et al. (2017) [73]	To examined the following research question: “Within primary care offices with high rates of patient-portal use, what do experienced physician and patient users of the ambulatory portal perceive as the benefits and challenges of portal use in general and secure messaging in particular?”	Qualitative	The USA	MyChart, an interactive patient portal allowed viewing demographics and test and lab results Capabilities: schedule appointments, request refills and send secure messages to providers.	Tethered	Adult patients with at least one cardiopulmonary condition	13 Family Medicine providers in the department of Family Medicine and 29 of their patients who had at least one chronic condition.	- Concerns about imposing on the physician and the lack of provider reimbursement for interactions - Uncertainty shared by both physicians and patients about how the patient should use the messaging function of PHR (e.g., lack of clarity about when to send a secure message; concern about unfocused or insufficient information in the messages, inappropriate message topics, and incorrect use of the secure messaging feature)
Cerdan et al. (2017) [40]	To gain insight into the experiences of patients with long-term conditions enrolled in an online rehabilitation program using a web portal.	Qualitative	Denmark	The Digital patient booklet, a patient portal for rehabilitation with supportive information and exercise programs for self-management activities	Not documented	Adults patients with heart disease, lymphedema and chronic pulmonary obstructive disease	Patients with heart disease, lymphedema and chronic pulmonary obstructive disease	- Patients preferred personal contact with physicians and physiotherapists - Technical issues regarding access, content, graphics and sounds, and terminology such as in non-native language - Negative attitudes towards PHR following negative attitudes to their received routine care and experiences of confusions, misunderstandings, and contradictory messages from providers regarding their diagnosis
Tieu et al. (2017) [77]	To examine specific usability barriers to patient portal engagement among a diverse group of patients and caregivers.	Mixed	The USA	Web-based PHR with links to online health education library; allows viewing visit summaries, prescribed health education, test results and looking up general health information	Tethered	Adult patients with diabetes, hypertension, asthma or COPD, heart disease, heart failure or chronic kidney disease	Patients with diabetes, hypertension, asthma or COPD, heart disease, heart failure or chronic kidney disease and their caregivers and 2 care providers	- Basic, health, and computer literacy challenges e.g., difficulty understanding non-health as well as medical terms and the interpretation of treatment plans and test results, inexperienced using search bars or uniform resource locators (URLs), difficulty while navigating the portal
Williamson et al. (2017) [85]	To characterize how young adult survivors and parent proxies of survivors <18 years old use a PHR	Quantitative	The USA	SurvivorLink, a web-based PHR allowing users to upload and store important health documents and electronically share these documents with their providers independent of institutional or practice specific electronic medical records systems	Stand alone	Pediatric cancer patients	Patients with cancer and their parents	- Black PHR registrants were significantly less likely to use the SurvivorLink in a meaningful way - Young adult registrants (>18 years old) or those who transitioned during the observation period were significantly more likely to use SurvivorLink in a meaningful way compared to those < 18 (with their proxy parent users).
Peremislov (2017) [63]	To explore electronic communication (e-communication or e-message encounter) between patients with type 2 diabetes and their providers within the patient portal.	Qualitative	The USA	A patient portal allowing patient-provider e-communication (no further details were documented)	Tethered	Adult type 2 diabetes patients	Patients with type 2 diabetes	- Of 71 e-communications initiated by providers, 49.2 % were from primary care physician (PCP) staff, 30.9% from PCP, 14.1% from care coordinators, 2.8% from pharmacists, 1.4% from diabetes clinic staff and 1.4% from specialty care team members
Price-Haywood et al. (2017) [65]	To examined the relationship between health literacy, portal use status, and interest in using websites or smartphone applications for tracking health information and to identify specific facilitators and barriers to use the portal.	Quantitative	The USA	^a^The patient portal of Epic systems called “MyOchsner” allowing patients to securely schedule/cancel non-urgent appointments, request medication refills, send and receive secure messages, view/download their health records, and access medical tools (e.g., wireless or patient-entered flow sheet data)	Tethered	Adult patients with hypertension and/or diabetes	247 patients with hypertension and/or diabetes	- Despite high rates of having access to computers, cell phones, and an Internet connection (>70%), portal nonusers most frequently cited preference for phone communication as the most common reason for not using the portal (75%). - Compared to nonusers, a higher proportion of users rated portal features useful. - e-health scores were positively associated with higher education and negatively associated with age. The odds of portal usage increased with total e-health score and decreased among black patients. The odds of being interested in using websites/smartphone apps increased with total e-health score - Portal nonusers mostly expressed concerns about online security of their information, lack of personalization in using technology, lack of resources, desire for skills or technical support to navigate computers and/or the Internet, and simply not seeing the need for or value of using the portal to manage their health. - There were concerns among users about computer literacy, the cumbersome nature of logging into portal accounts (e.g., remembering passwords, multiple accounts for patients in the same household), lack of technical support, and variations in provider availability for online appointment scheduling and response times to medical messages - Patient Research Advisory Board identified a lack of a clear tangible incentives for using the portal as a supplement to the traditional provider-patient relationship as a major area of concern for portal nonusers
Price-Haywood et al. (2018) [66]	To examine whether the intensity of bidirectional secure portal messaging is associated with improved clinical outcomes.	Quantitative	The USA	^a^The patient portal of Epic systems called “MyOchsner” allowing patients to securely schedule/cancel non-urgent appointments, request medication refills, send and receive secure messages, view/download their health records, and access medical tools (e.g., wireless or patient-entered flow sheet data)	Tethered	Adult patients with hypertension or diabetes	Patients with hypertension or diabetes	- A higher proportion of patients who were age 50 years and older, female, white non-Hispanic, and with co-morbid diabetes and hypertension had higher frequency and intensity of medical advice messaging - Compared to portal nonusers, portal users were younger, and a higher proportion were female, lived in zip code regions with higher average household incomes, and were commercially insured. Among portal users there were a lower proportion of black non-Hispanic patients, and lower average Charlson comorbidity scores
Ali et al. (2018) [33]	To identify task-technology fit problems and usability challenges in the novel portal, recommend solutions, and to evaluate whether the recommended design changes improved usability	Mixed	The USA	“myNYP” (New York Presbyterian), an electronic patient portal providing patients with inpatient data such as laboratory results, procedures, and care instructions after their hospital discharge	Tethered	Adults chronic patients	23 participants which consisted of patients with chronic conditions including types I and II diabetes, and cancer, and also caregivers caring for family members with conditions such as ulcerative colitis and thalassemia	- A number of usability barriers (e.g., failure to use users’ language and insufficient guidance on the portal) - Mismatch between users, tasks and technology (e.g., lack of a very concrete understanding of health information management tasks - Problems of consolidating data and medical records scattered across multiple doctors and sharing them
van den Heuvel et al. (2018) [80]	Primary objectives: To test the feasibility of a PHR for bipolar patients To evaluate the user experiences of persons with bipolar disorders (BD) involving informal caregivers, and clinicians. The secondary objective: To examine changes in quality of life, empowerment, symptom reduction, changes in mood and activity, and illness burden and severity.	Quantitative	The Netherlands	A Web-based online personal health record Allowing to view medical record, medication, treatment, and medical passport, laboratory results and reports, mood chart, general information about the features of BD Capabilities: a personal messages module to communicate with the appointed clinician and a personal crisis plan from interpretation of mood chart	Untethered	Adult bipolar disorder patients	66 patients with diagnosis of bipolar disorders and eleven clinicians (e.g., psychiatrists, advanced nurse practitioners, and community psychiatric nurses)	- Over a third of the clinicians favored direct telephone contact instead of communication through PHR due to concern that electronic communications distorted open communication and decreased the responsibility of the participant with BD to get in contact for appropriate help - From those patient participants who not responded at study's endpoint, 81.5% gave the following reasons for dropping-out: the PHRBD was too much work, they did not perceive the added value, were too busy, and felt too confronted when monitoring the course of their illness - Some clinicians (32.1%) disagreed with informal caregiver access to PHR due to concerns about impairing the privacy of the patient, might be patronizing, and might complicate communication between partners about the illness at a premature stage. - A lack of compatibility with existing hospital electronic medical record systems by using the messages function (e.g., need to copy and paste reports from the records system into the PHR-BD and vice versa, which was considered too time-consuming and unfeasible for daily practice)
Latulipe et al. (2018) [53]	To examine how older adult patients perceive the benefits and risks of proxy patient portal access by their caregivers.	Qualitative	The USA	A patient portal (no details available)	Not documented	Adult patients with diabetes, hypertension, dyslipidemia, or cardiovascular disease	10 patients with diabetes, hypertension, dyslipidemia, or cardiovascular disease	- Concerns about the privacy of their information when a stigmatized condition existed or the confidentially of their financial information
Nahm et al. (2018) [56]	To examine the current state of older chronic patients” patient portal use and their experiences with patient portal training	Mixed	The USA	At least 38 different patient portals across the country	Not applicable	Adult patients with at least one chronic disease including hypertension, arthritis, depression, and others	Patient with at least one chronic disease including hypertension, arthritis, depression, and others	- Participants’ level of knowledge and self-efficacy for PPs were relatively low with an average PP knowledge of 5.2 ± 1.7 and the mean self-efficacy for PP use of 27.1 ± 11.9 - Having multiple portals from multiple providers and not remembering which one was from which provider - Participants’ perceived usability of their portals (primary if they had multiple) was low, with a mean of 28.7
Powell and Myers (2018) [64]	To explore how patients are introduced to and learn about portals and how patients and providers perceive the usefulness of a portal in the context of chronic illness self-management.	Qualitative	The USA	Web based electronic patient portals in general	Not applicable	Adult patients with multiple chronic conditions (diabetes, hypertension, heart disease, or coronary artery disease)	9 patients and 7 healthcare providers	- Difficulty accessing the portal due to passwords, computer, or server problems identified as a barrier by both patients and providers - Unavailable functions such as correcting errors in records or changing the preferred pharmacy - Many patients and providers described their preference for interacting with a person rather than via the portal - A number of provider-specific barriers with three subcategories: lack of time, payment concerns, and regulatory barriers - Multiple providers mentioned the need for payment reform, specifically capitated payments, so that providers could be compensated for their work via the portal.

*Abbreviations*: *PHR* personal health records, *EMR* electronic medical record, *EHR* electronic health record, *CKD* chronic kidney disease, *OR* Odd Ratio, *COPD* chronic obstructive pulmonary disease, *BD* bipolar disorders, *HIV* human immunodeficiency virus, *MGP* my health profile, *the USA* the United States of America, *the UK* the United Kingdom

^a^data was completed using the authors’ another publication i.e., “Primary Care Practice Reengineering and Associations With Patient Portal Use, Service Utilization, and Disease Control Among Patients With Hypertension and/or Diabetes”; Ochsner J. 2017 Spring; 17(1): 103–111

^b^In the chronological order of publication year

### The methodological quality of the included studies

Additional file 3 provides the results of the quality check for included studies. There were some quality issues mainly about data collection and interpretation in four studies [5, 87–89]. Because the MMAT discourages excluding studies based on methodological quality, we did include all 60 identified studies in our analysis and report.

## Discussion

Understanding barriers that prevent realizing the ePHR’s full benefits is a prerequisite to future work aimed at its optimal use. Our comprehensive review identified 60 relevant studies, which reported barriers to ePHR adoption/use associated with the interacting factors of personal, environmental/medical practice, technology, and chronic disease condition. Our findings expand on those of earlier reviews [18, 19] and point out that our knowledge base for this topic is still limited (and one dimensional), with most of the research predominantly focusing on facilitators than barriers and also on barriers at the patient level than those existed beyond the patient level in chronic disease care.

Differences among users of health information technology (HIT) and the implication of their needs and requirements for design and development have recently gained further attention [91–94]. ePHRs are aiming to empower *patients* and/or *caregivers* and engage them in collaborative and productive chronic care with *health professionals*. Failure to acknowledge the characteristics, needs, and requirements of all these user groups will lead to the development of unpredictable barriers to ePHR adoption leading to its sub-optimal use. In line with the previous literature, our findings highlight the impact of “digital divide” at the patient level (in terms of age, gender, health, and technology literacy, and socioeconomic status) and several attitudinal factors such as coping styles with a chronic condition (e.g., denial of a condition) and preferences for personal communications with care providers (e.g., preference for direct contact) [19, 95, 96]. Our review also points out that the literature has heavily focused on the elderly, probably because they are disproportionately represented among patients with chronic diseases. Thus, it is plausible that the barriers faced by the younger and also middle-aged (<50 years old) chronic patients would be underrecognized and ePHR use in these groups may fall behind. This is particularly important because of the increasing prevalence of chronic diseases such as diabetes in these age groups. These groups increasingly represent users with higher educational levels and technology literacy (with higher needs and expectations), compared with that of the elderly, introducing a niche market for ePHRs and a unique opportunity to tap into their potential. Moreover, while providing the care for young chronic patients (e.g., between 10 and 19 years old), these adolescent users are a different user group when compared to their parent caregivers and this becomes more important especially when these patients transit from pediatric care to receive adulthood care. Thus, their needs and requirements for an effective and useful ePHR should be given much attention helping a smooth and safe care transition. Such an approach will, to a great extent, make sure that using an ePHR becomes an enriching experience for both the adolescents and those involved in their care.

Most of the healthcare systems have important constraints in terms of human resources shortages, inadequate infrastructure, and insufficient finances, which require mindful management to operate and maximize efficiency [97]. ePHRs have the potentials to do so by facilitating self-care and virtual visits. However, in the context of ePHR use, the responsibilities of patients and providers are changed in many ways. For example, they need to make sure that the data available in different locations are accurate, integrated, and updated [3]. This is important particularly because data about chronic care is scattered throughout different EHRs that do not speak together; and then, the task of data integration is informally delegated to patients. ePHRs can be used meaningfully if they are implemented as a component (i.e., a tool for self-management) of comprehensive care models developed for chronic care (such as the Wagner’s Chronic Care Model [98]). If such models are implemented and proper links are made among their components (i.e., self-management, clinical information systems, disease registries, and decision support systems), patients are freed from extra responsibilities and can focus on productive “self-management” through an ongoing collaborative process with their providers via ePHRs. Therefore, as the adoption of ePHRs are very related to the adoption of EHRs, the barriers related to EHRs in the first place and then the interoperability between these two should adequately be addressed [17, 21, 27]. For example, providers should consider ePHRs’ potentials and their fit within the information infrastructure of their practice when they commence investing in EHRs and choosing their vendors. This becomes especially important after changes that the outbreak of the novel coronavirus disease has brought up to the current and future practice in terms of managing virtual visits. Unfortunately, discussion on such issues has been underrepresented in the identified studies, which should be taken into account in future research.

Our review shows that the barriers related to the providers and the organization of chronic care have not fully been studied despite their importance (studied by only 8 studies). The lack of provider interest and even their resistance to adopting ePHRs are important [3, 12, 21, 99]. Provider concerns about the impacts on workload, professional/legal liabilities, relationships with patients, and the lack of reimbursements should be fully addressed [15, 21, 100, 101]. Moreover, their involvement in ePHR use has not been given full attention as it deserves. In a review of 19 ePHRs, only half had enabled user actions taken by *physicians* [17]. Providers can act as an effective catalyst in this regard by practicing their social influence on patients [102]. Scholars have highlighted that without involving providers in ePHR’s design, implementation, and application and without addressing their barriers, efforts for widespread ePHR adoption/use would be in vain [3, 103, 104]. Therefore, it will be insightful if future studies explore in more depth provider issues and how they can further be engaged with this emerging technology in chronic care.

Functionalities of ePHRs that provide solutions for personalized needs and requirements of chronic patients have important implications for their adoption and use, as also emerged in our review [17, 19, 73, 90, 105]. One review suggested that features such as access to personal health data and general health information, communicating with providers and support groups, and receiving personal decision support were linked to empirical evidence of benefits from ePHR-enabled self-management [19]. Yet, no ePHR in that review described a platform for all those features. Furthermore, the necessity for measures to ensure the privacy and confidentiality in record transactions and communication through ePHRs was a serious concern voiced by clinical directors and health information technology leaders, besides patients [10, 12, 45, 47, 52, 58, 65, 68, 72, 76, 78, 80]. The relevancy of this concern has also been highlighted elsewhere [20, 21, 27, 95, 106]. Reviewing privacy policies of 24 ePHRs showed that such concerns are very relevant and that compliance with privacy standards and regulations were generally low [14]. It has been recommended that institutions should assemble governance groups to develop policies regarding security, privacy, and confidentiality of records to assure ePHR users on preserving their rights [12].

### Strengths and weaknesses of our review

To our knowledge, no study to date has analyzed ePHR studies exclusively concerning barriers to its adoption and use in *chronic care*. Nevertheless, our review has several limitations. First of all, we only included studies published in English. Second, facilitators and barriers to the adoption of technology is a complex concept without an agreed-upon research methodology. It is plausible that many of the discussions about these core concepts have appeared only in non-peer-reviewed or research publications such as white papers, perspectives, editorials, etc. The findings of our systematic review are confined by the content of included articles, and hence may not well reflect a proper balance of what is known on the topic. Such reviews, however, point out the gaps and direct future studies. Third, ePHRs are an evolving technology with new features and functionalities and so is their position in chronic care. Therefore, the barriers identified in this review are possibly not generalizable to all patient populations or different implementation strategies and healthcare systems. For example, a majority of studies are from the US and therefore a Western viewpoint is predominant here. Therefore, it should be born in mind that the barriers faced by users might be different in different healthcare contexts.

## Conclusion

If we are to reap the full benefits of ePHRs in chronic care, we ought to understand the unique characteristics of this type of care and the barriers and challenges that ePHR users face in adoption and sustained use, in the first place. This knowledge should be used to make ePHR functionalities that fit in these unique characteristics well. Future research must aim at identifying the barriers experienced especially by younger chronic patients and their requirements and expectations, and also those barriers faced by care providers all beyond the patient level. A deeper understating of these barriers will reveal opportunities that if addressed in the design, development, and implementation can lead to the enhanced use of ePHRs.

## Supplementary information

**Additional file 1:** The search strategy in the electronic databases used in our study.

**Additional file 2:** The main reasons for exclusion of articles.

**Additional file 3:** Quality of included studies by the MMAT tool.

**Additional file 4.** Studies providing information on barriers to PHR adoption and use in chronic care.

## Data Availability

All data generated or analyzed during this systematic review are included in this published article [and its supplementary information files].

## Abbreviations

ePHRs: Electronic Personal Health Records
CINAHL: Cumulative Index to Nursing and Allied Health Literature
IEEE: Institute of Electrical and Electronics Engineers
HITECH: Health Information Technology for Economic and Clinical Health Act
EHRs: Electronic Health Records
US: The United States
PRISMA: Preferred Reporting Items for Systematic reviews and Meta-Analyses
MMAT: Mixed Methods Appraisal Tool
PHRAM: Personal Health Records Adoption Model
UTAUT: Unified Theory of Acceptance and Use of Technology
HIT: Health Information Technology
UUMS: Urmia University of Medical Sciences
