# Supplementary material for: Barriers to patient, provider, and caregiver adoption and use of electronic personal health records in chronic care: a systematic review
Source: BMC Med Inform Decis Mak. 2020 Jul 8;20:153. doi: 10.1186/s12911-020-01159-1 (PMC7341472; doi:10.1186/s12911-020-01159-1)
Supplement: Supplementary file 1 — Additional file 1: The search strategy in the electronic databases used in our study. [file 12911_2020_1159_MOESM1_ESM.docx]

**Appendix 1.** The search strategy in the electronic databases used in our study

| Database | Search Strategy |
| --- | --- |
| PubMed | 1#: (chronic diseases OR Disease*, chronic OR chronic Illness* OR Illness*, chronic OR chronically ill OR chronic condition)  2#: (health record, personal OR personal health record OR Record* , personal health OR personal health records OR personal health information OR health information, personal OR information, personal health OR personal medical records OR medical Record* , personal OR personal medical record OR Record* , personal medical OR patient portals OR patient web portal OR Portal* , patient web OR Web Portal* , patient OR patient internet portals OR internet Portal* , patient OR patient internet portal OR Portal* , patient internet OR patient web portals OR patient portal OR portal, patient OR PHR OR e-phr OR personal electronic health records OR electronic personal health records OR patient portal OR patient-held record)  3#: English[language]  4#: ("2005"[Date - Publication]: "2018"[Date - Publication])  5#: review [Publication Type]  6#: 1# AND 2# AND 3# AND #4 NOT 5# |
| Ovid MEDLINE | 1#: ((Health Record, Personal or Personal Health Record or Record*, Personal Health or Personal Health Records or Personal Health Information or Health Information, Personal or Information, Personal Health or Personal Medical Records or Medical Record*, Personal or Personal Medical Record or Record*, Personal Medical or Patient portals or Patient Web Portal or Portal*, Patient Web or Web Portal*, Patient or Patient Internet Portals or Internet Portal*, Patient or Patient Internet Portal or Portal*, Patient Internet or Patient Web Portals or Patient Portal or Portal, Patient or PHR or e-PHR or personal electronic health records or electronic personal health records or patient portal or patient-held record) and (Chronic Diseases or Disease*, Chronic or Chronic Illness* or Illness*, Chronic or Chronically Ill or chronic condition)).mp. [mp=title, abstract, full text, caption text]  2#: yr="2005 - 2018"  3#: original articles  4#: 1# AND 2# AND 3# |
| \| \| Science Direct \| \| --- \| \| \| --- \| --- \| | 1#: Title, abstract or keywords (Personal Health Information OR Personal Medical Records OR Patient portals OR Patient Web Portal OR Patient Internet Portals OR e-PHR OR personal electronic health records OR patient-held record)  2#: Title, abstract or keywords (Chronic Diseases OR Disease*, Chronic)  3#: Title NOT review  4#: Year (2005-2018)  5#: Article Types (Research articles)  6#: 1# AND 2# AND 3# AND 4# AND #5 |
| CINAHL | 1#: TX (Health Record, Personal or Personal Health Record or Record*, Personal Health or Personal Health Records or Personal Health Information or Health Information, Personal or Information, Personal Health or Personal Medical Records or Medical Record*, Personal or Personal Medical Record or Record*, Personal Medical)  2#: TX (Chronic Diseases or Disease*, Chronic or Chronic Illness* or Illness*, Chronic or Chronically Ill or chronic condition)  3#: 1# AND 2# |
| IEEE explore | 1#patient Web Portal OR Patient portals OR Health Record, Personal OR Personal Health Record OR Personal Medical Records  2#: (Chronic Diseases OR Disease*, Chronic OR Chronic Illness*)  3#: Publication Year: 2005-2018  4#: "Publication Title": review  5#: 1# AND 2# AND 3# NOT 4# |
| Cochrane trials | health record, personal OR personal health record OR Record* , personal health OR personal health records OR personal health information OR health information, personal OR information, personal health OR personal medical records OR medical Record* , personal OR personal medical record OR Record* , personal medical OR patient portals OR patient web portal OR Portal* , patient web OR Web Portal* , patient OR patient internet portals OR internet Portal* , patient OR patient internet portal OR Portal* , patient internet OR patient web portals OR patient portal OR portal, patient OR PHR OR e-phr OR personal electronic health records OR electronic personal health records OR patient portal OR patient-held record) AND (chronic diseases OR Disease*, chronic OR chronic Illness* OR Illness*, chronic OR chronically ill OR chronic condition) |
