# Supplementary material for: Barriers to patient, provider, and caregiver adoption and use of electronic personal health records in chronic care: a systematic review
Source: BMC Med Inform Decis Mak. 2020 Jul 8;20:153. doi: 10.1186/s12911-020-01159-1 (PMC7341472; doi:10.1186/s12911-020-01159-1)
Supplement: Supplementary file 2 — Additional file 2: The main reasons for exclusion of articles. [file 12911_2020_1159_MOESM2_ESM.docx]

Appendix B: The main reasons for exclusion of articles

Phase 1: screening titles and abstracts

- Non-English publications
- Intervention was not an electronic PHR/patient portal (e.g., registries, mobile health technologies, decision support systems, tele-monitoring devices, surveillance systems, educational websites, personal websites, paper-based personal health records, m-health, assistive living, etc).
- Aims of studies was out of the scope of our review (e.g., evaluating the effects of medications and their side effects or other medication management issues, virtual realities, etc.)
- Evaluating the impact of disease on chronic patients’ life
- Evaluating the epidemiology of chronic diseases
- Review articles, opinion papers, editorials, letter to editors
- Abstracts presented in a congress

Phase 2: full text reviews

- Chronic patients were a minority of an study's general population
- Paper-based PHR
- Evaluation of PHRs in combination with other HIT such as health information and diabetes self-management websites, mobile health applications
- Design or implementation reports
- Not an evaluation study of a PHR (e.g., a case report without a study design)
- Not an evaluation study of a PHR's barriers
- Study of patients' clinical outcomes
- Study of a solution for a barrier not the barrier itself
