# Supplementary material for: Barriers to patient, provider, and caregiver adoption and use of electronic personal health records in chronic care: a systematic review
Source: BMC Med Inform Decis Mak. 2020 Jul 8;20:153. doi: 10.1186/s12911-020-01159-1 (PMC7341472; doi:10.1186/s12911-020-01159-1)
Supplement: Supplementary file 3 — Additional file 3: Quality of included studies by the MMAT tool. [file 12911_2020_1159_MOESM3_ESM.docx]

**SUPPLEMENTARY (APPENDIX C)**

**Quality of qualitative studies**

Abbreviations:

Y=Yes, N=No, C= Can't tell

| Title | Are there clear research questions? | Do the collected data allow to address the research questions? | Is the qualitative approach appropriate to answer the research question? | Are the qualitative data collection methods adequate to address the research question? | Are the findings adequately derived from the data? | Is the interpretation of results sufficiently substantiated by data? | Is there coherence between qualitative data sources, collection, analysis and interpretation? | Comments |
| --- | --- | --- | --- | --- | --- | --- | --- | --- |
| Personal Electronic Health Records: Understanding User Requirements and Needs in Chronic Cancer Care | Y | Y | Y | Y | Y | Y | Y |  |
| Parents' Perceptions of a Patient Portal for Managing Their Child's Chronic Illness | Y | Y | Y | Y | C | Y | Y | The research presented in a brief format which hinders further in depth evaluation of its quality |
| Influencing factors for adopting personal health record (PHR) | Y | Y | Y | C | C | Y | C |  |
| e-Patients Perceptions of Using Personal Health Records for Self-management Support of Chronic Illness | Y | Y | Y | Y | Y | Y | Y |  |
| Exploring Challenges and Potentials of Personal Health Records in Diabetes Self-Management: Implementation and Initial Assessment | Y | Y | Y | Y | Y | Y | Y |  |
| Barriers to the use of a Personal Health Record by an Elderly Population | Y | N | Y | N | N | N | N | The qualitative methods of data collection and analysis was not sufficiently explored |
| Using Health Information Technology to Foster Engagement: Patients’ Experiences with an Active Patient Health Record | Y | Y | Y | Y | Y | Y | Y |  |
| Patients Know Best: Qualitative Study on How Families Use Patient-Controlled Personal Health Records | Y | Y | Y | Y | Y | Y | Y |  |
| The Rules of Engagement: Perspectives on Secure Messaging From Experienced Ambulatory Patient Portal Users | Y | Y | Y | Y | Y | Y | Y |  |
| Barriers and Facilitators to Online Portal Use Among Patients and Caregivers in a Safety Net Health Care System: A Qualitative Study | Y | Y | Y | Y | Y | Y | Y |  |
| Exploring Three Perspectives on Feasibility of a Patient Portal for Older Adults. | Y | C | Y | C | C | N | N |  |
| Online communication in a rehabilitation setting: Experiences of patients with chronic conditions using a web portal in Denmark | Y | Y | Y | Y | Y | Y | Y |  |
| Integrating the patient portal into the health management work ecosystem: user acceptance of a novel prototype | Y | Y | Y | C | C | Y | Y |  |
| A Shared e-Decision Support Portal for Pediatric Asthma | Y | Y | Y | Y | C | Y | Y |  |
| Design Considerations for Patient Portal Adoption by Low- Income, Older Adults | Y | Y | Y | Y | Y | Y | Y |  |
| Insights Into Older Adult Patient Concerns Around the Caregiver Proxy Portal Use: Qualitative Interview Study | Y | Y | Y | Y | Y | Y | Y |  |
| Patient and Parent Views on a Web 2.0 Diabetes Portal—the Management Tool, the Generator, and the Gatekeeper: Qualitative Study | Y | Y | Y | N | Y | Y | Y |  |
| Patient Use of the Electronic Communication Portal in Management of Type 2 Diabetes | Y | Y | Y | Y | Y | Y | Y |  |
| Electronic Patient Portals: Patient and Provider Perceptions | Y | Y | Y | Y | C | Y | Y |  |
| Implementing and using a patient portal: a qualitative exploration of patient and provider perspectives on engaging patients | Y | C | Y | Y | C | Y | Y |  |
| Electronic Personal Health Records for Childhood Cancer Survivors: An Exploratory Study | Y | Y | Y | Y | Y | Y | Y |  |
| Improving Diabetes Management With a Patient Portal: Qualitative Study of a Diabetes Self-Management Portal | Y | Y | Y | Y | Y | Y | Y |  |
| Incorporating Patient Perspectives into the Personal Health Record: Implications for Care and Caring | Y | Y | Y | Y | Y | Y | Y |  |
| Interest in the Use of Computerized Patient Portals: Role of the Provider–Patient Relationship | Y | Y | Y | Y | Y | Y | Y |  |
| Core Features of a Parent-controlled Pediatric Medical Home Record | Y | N | Y | N | C | N | N |  |

**Quantitative randomized studies**

| Title | Are there clear research questions? | Do the collected data allow to address the research questions? | Is randomization appropriately performed? | Are the groups comparable at baseline? | Are there complete outcome data? | Are outcome assessors blinded to the intervention provided? | Did the participants adhere to the assigned intervention? | Are the participants representative of the target population? | comments |
| --- | --- | --- | --- | --- | --- | --- | --- | --- | --- |
| Internet portal use in an academic multiple sclerosis center | Y | Y | Y | Y | Y | C | Y | Y |  |
| Personal health records and hypertension control: a randomized trial | Y | Y | Y | Y | Y | Y | Y | Y |  |

**Nonrandomized studies**

| Title | Are there clear research questions? | Do the collected data allow to address the research questions? | Are the participants representative of the target population? | Are measurements appropriate regarding both the outcome and intervention (or exposure)? | Are there complete outcome data? | Are the confounders accounted for in the design and analysis? | During the study period, is the intervention administered (or exposure occurred) as intended? | Comments |
| --- | --- | --- | --- | --- | --- | --- | --- | --- |
| The user experiences and clinical outcomes of an online personal health record to support self-management of bipolar disorder: A pretest-posttest pilot study | Y | Y | Y | Y | Y | N | N |  |
| Differences Between Diabetes Patients Who Are Interested or Not in the Use of a Patient Web Portal | Y | Y | Y | Y | Y | C | Y |  |

**Quantitative descriptive**

| Title | Are there clear research questions? | Do the collected data allow to address the research questions? | Is the sampling strategy relevant to address the research question? | Is the sample representative of the target population? | Are the measurements appropriate? | Is the risk of nonresponse bias low? | Is the statistical analysis appropriate to answer the research question? | Comments |
| --- | --- | --- | --- | --- | --- | --- | --- | --- |
| Perceptions of chronically ill and healthy consumers about electronic personal health records: a comparative empirical investigation | Y | Y | Y | Y | Y | Y | Y |  |
| Electronic personal health record use among registered nurses | Y | Y | Y | Y | Y | Y | Y |  |
| Patients with chronic kidney disease and their intent to use electronic personal health records | Y | C | C | C | Y | C | Y | RR and sampling methods were not reported. |
| Challenges to Using an Electronic Personal Health Record by a Low-Income Elderly Population | Y | Y | Y | Y | Y | N | Y | The socioeconomic condition as well as other problems in access and use of Information technology increased the risk of nonresponse. |
| An exploratory study of the personal health records adoption model in the older adult with chronic illness | Y | Y | N | N | C | C | Y |  |
| Integrated Personal Health Record Use: Association With Parent-Reported Care Experiences | Y | Y | Y | Y | Y | Y | Y |  |
| Patient Portal Utilization Among Ethnically Diverse Low Income Older Adults: Observational Study | Y | Y | C | C | Y | N | Y |  |
| The Digital Divide and Patient Portals: Internet Access Explained Differences in Patient Portal Use for Secure Messaging by Age, Race, and Income | Y | Y | Y | Y | Y | Y | Y |  |
| Predictive variables of the Use of Personal Health Record: the Hospital Italiano de Buenos Aires Study | Y | N | N | N | C | C | Y |  |
| eHealth Literacy: Patient Engagement in Identifying Strategies to Encourage Use of Patient Portals Among Older Adults | Y | Y | Y | Y | Y | Y | Y |  |
| Patient Perceptions of a Personal Health Record: A Test of the Diffusion of Innovation Model | Y | Y | Y | Y | Y | Y | Y |  |
| Dose effect of patient–care team communication via secure portal messaging on glucose and blood pressure control | Y | Y | Y | Y | Y | Y | Y |  |
| The Literacy Divide: Health Literacy and the Use of an Internet- Based Patient Portal in an Integrated Health System—Results from the Diabetes Study of Northern California (DISTANCE) | Y | Y | Y | Y | Y | Y | Y |  |
| Social disparities in internet patient portal use in diabetes: evidence that the digital divide extends beyond access | Y | Y | Y | Y | Y | Y | Y |  |
| Disparities in registration and use of an online patient portal among older adults: findings from the LitCog cohort | Y | Y | Y | Y | Y | Y | Y |  |
| The Association Between Personal Health Record Use and Diabetes Quality Measures | Y | Y | Y | Y | Y | Y | Y |  |
| Use of a Shared Medical Record With Secure Messaging by Older Patients With Diabetes | Y | Y | Y | Y | Y | Y | Y |  |
| Meaningful Use of an Electronic Per- sonal Health Record (ePHR) among Pediatric Cancer Survivors | Y | Y | Y | Y | Y | Y | Y |  |
| Patient–provider communication and trust in relation to use of an online patient portal among diabetes patients: The Diabetes and Aging Study | Y | Y | Y | Y | Y | Y | Y |  |

**Mixed-methods**

| Title | Are there clear research questions? | Do the collected data allow to address the research questions? | Is there an adequate rationale for using a mixed methods design to address the research question? | Are the different components of the study effectively integrated to answer the research question? | Are the outputs of the integration of qualitative and quantitative components adequately interpreted? | Are divergences and inconsistencies between quantitative and qualitative results adequately addressed? | Do the different components of the study adhere to the quality criteria of each tradition of the methods involved? | Comments |
| --- | --- | --- | --- | --- | --- | --- | --- | --- |
| Focus Section Health IT Usability: Applying a Task-Technology Fit Model to Adapt an Electronic Patient Portal for Patient Work | Yes | Yes | Yes | Yes | Yes | yes | yes |  |
| Usability Testing Finds Problems for Novice Users of Pediatric Portals | Y | Y | Y | Y | C | C | C | This study's data collection and analysis of qualitative section were briefly explored and enough information was not provided |
| Family perceptions of the usability and value of chronic disease web- based patient portals | Y | Y | Y | Y | Y | C | C | Qualitative part of the study was described very briefly. |
| Barriers and Facilitators of Online Patient Portals to Personal Health Records Among Persons Living With HIV: Formative Research | Y | Y | Y | N | N | N | N | The study used focus group to complement their quantitative study but it reported the qualitative methodology and result sections very briefly |
| Family attitudes towards an electronic personal health record in a long term care facility | Y | Y | Y | Y | Y | Y | Y |  |
| Exploring Factors Related to the Adoption and Acceptance of an Internet-based Electronic Personal Health Management Tool (EPHMT) in a Low Income, Special Needs Population of People Living with HIV and AIDS in New York City | Y | Y | Y | Y | Y | Y | Y |  |
| Meeting the health information needs of prostate cancer patients using personal health records | Y | Y | Y | Y | Y | Y | Y |  |
| Online patient websites for electronic health record access among vulnerable populations: portals to nowhere? | Y | Y | Y | Y | Y | Y | Y |  |
| Organizational strategies for promoting patient and provider uptake of personal health records | Y | Y | Y | Y | C | Y | Y |  |
| Patient reported barriers to enrolling in a patient portal | Y | Y | Y | N | Y | N | Y |  |
| Barriers to patient participation in a self-management and education website Renal PatientView: A questionnaire-based study of inactive users | Y | Y | Y | C | Y | N | N |  |
| Patient Portal Use Among Older Adults: What Is Really Happening Nationwide? | Y | Y | Y | Y | Y | Y | Y |  |
| Processes and outcomes of developing a continuity of care document for use as a personal health record by people living with HIV/AIDS in New York City | N | C | C | C | C | N | C |  |
| Understanding Patient Portal Use: Implications for Medication Management | Y | Y | Y | Y | Y | N | Y |  |
| Secure messaging and diabetes management: experiences and perspectives of patient portal users | Y | Y | Y | Y | Y | Y | Y |  |
