# Supplementary material for: Barriers to patient, provider, and caregiver adoption and use of electronic personal health records in chronic care: a systematic review
Source: BMC Med Inform Decis Mak. 2020 Jul 8;20:153. doi: 10.1186/s12911-020-01159-1 (PMC7341472; doi:10.1186/s12911-020-01159-1)
Supplement: Supplementary file 4 — Additional file 4. Studies providing information on barriers to PHR adoption and use in chronic care. [file 12911_2020_1159_MOESM4_ESM.docx]

Table 1. Studies providing information on barriers to PHR adoption and use in chronic care

| **Authors (year of publication) ^[[1]](#footnote-1)^** | **Study objective** | **Research method** | **Country** | **Features of PHRs (if any)** | **Integration with EMR/EHR** | **PHR's target chronic patient population** | **Study participants** | **Main outcomes in relation to the objective of the current study** |
| --- | --- | --- | --- | --- | --- | --- | --- | --- |
| Lober et al. (2006) ([1](#_ENREF_1)) | To evaluate the barriers faced by a low income, elderly population in creating and using a personal health record. | Qualitative | The USA | A Personal Health Information Management System (PHIMS), allows viewing personal demographics, past surgeries and immunization records, environmental factors and foods, medications and allergies to medications, also with capabilities of messaging with provider, sharing printed version of information with providers or family | Untethered | Adults elderly patients | 38 elderly residents of a nursing home, many had chronic disease | - Health and computer literacy and anxiety  - Physical and cognitive impairments of elderly  - Problems related to access to the computers (e.g., not owning a computer) and access to an assistant to use the system (e.g., availability of nurses or social workers) |
| Hess et al. (2007) ([2](#_ENREF_2)) | To explore challenges to  office-based implementation of a patient portal and initial patient reaction to the technology in the context  of diabetes care | Qualitative | The USA | University of Pittsburgh Medical Center (UPMC) HealthTrak (a patient portal) allowing to view test results, medication and problem lists, and health reminders, secure, electronic communication with the physician’s office, to view and schedule appointments, and disease-specific tools and information plus self-management tools for weight and blood pressure monitoring | Tethered | Adults diabetic patients | Diabetic patients | - Patient identified inefficiencies including missing lab results and radiology reports, inaccurate information, and slow responses from the physician and/or nurse.  - Barriers to use, including lost or unknown user names and passwords, being unaware of the features of the HealthTrak, not possible to contact all of patients' physicians, not just their primary care physician, due to lack of coordination and integration |
| Zickmund et al. (2008) ([3](#_ENREF_3)) | To discern the impact of the provider–patient relationship on interest in using a web-based patient portal | Qualitative | The USA | “HealthTrak”, a patient portal originally offered online information, laboratory results, and an encrypted and secure method for e-mailing messages. The enhanced version for diabetes patients allowed them to track glucose, blood pressure, and physical activity records online entered by them | Tethered | Adults diabetes patients | Patients with diabetes | - Disinterest in portal use was linked to patient satisfaction with the patient–provider relationship, so as participants with a satisfying provider–patient relationship appeared less in need of the patient portal.  - Barriers to learning the system such as lower computer literacy and the time required to learn  - Fear of losing personal communication with their primary physicians through emails (outside of the portal functionality)  - Concern about who in the office would be reading the e-mail messages sent over the portal because of the indirect routing of the e-mails sent through portal |
| Britto et al. (2009) ([4](#_ENREF_4)) | To evaluated the usability of “MyCare Connection” portal for parents of children with cystic fibrosis, diabetes or arthritis. | Mixed | The USA | Web-based secured web application allowing to view demographic and contact information; laboratory, radiology and pathology reports; inpatient and outpatient encounters; medications; and secure electronic messaging | Tethered | Children with cystic fibrosis, diabetes or arthritis | Parents of children with cystic fibrosis, diabetes or arthritis | - Clarity of information and the ability to comprehend error messages was scored least in the satisfaction study  - A number of problematic usability issues including: use of medical jargons and terminology; problematic clarity of normal and abnormal values; information overload and information complexity requiring medical interpretations and explanations; more help options and bolder and more eye-catching sidebars and instructions were needed |
| Kim et al. (2009) ([5](#_ENREF_5)) | To assess the use and utility of PHRs in a low-income, elderly population | Quantitative | The USA | A stand alone, individually controlled, Web-based repository of personal health information allowing users to enter, update, or delete structured information in nine different categories. It provides summary pages that list all the information entered into the system by the user. A hardcopy and/or electronic copy can be shared with health care providers or family members. | Untethered | Adults elderly chronic patients | Elderly residents of a nursing home with chronic diseases | - Most (77%) of the system use happened while assistance from graduate nursing students or housing staff was available to the residents. |
| Sarkar et al. (2010) ([6](#_ENREF_6)) | To examine use of an internet-based patient portal among a well characterized cohort of English-speaking adult patients with diabetes differed between those who report limited health literacy versus those who do not. | Quantitative | The USA | An internet based patient portal allows viewing laboratory test results, sending email to providers, requesting medication refills, and making medical appointments. | Tethered | Adults diabetes patients | Diabetes patients | - African-America, Latino, and Filipino race/ethnicities and lower educational attainments were associated with increased risk of not signing on to the patient portal.  -Those with limited health literacy had higher odds of never signing on to the patient portal |
| Weppner et al. (2010) ([7](#_ENREF_7)) | To Evaluate use of a web-based shared medical record (SMR) between older patients with diabetes and providers. | Quantitative | The USA | A web-based shared medical record allowing a secure messaging with health care providers, request medication re-fills and in-person appointments; and view test results, after-visit summaries, medical problem lists, allergies, and immunizations. | Tethered | Adults diabetes patients | Diabetes patients | - Unadjusted analyses indicated that younger age, male sex, living in a higher socio-economic neighborhood, and primary care physician level of secure messaging were associated with patients’ initial portal use  - Higher morbidity of patients was linked to higher signing up and continued use of the system |
| Wagner et al. (2010) ([8](#_ENREF_8)) | To examine patient perspectives on ePHR use and functionality as part of the development process of an existing ePHR | Qualitative | The USA | My HealthLink, an ePHR enabled consumers to store personal health information with core functions of secure messaging; access to educational materials; medication interaction checking; recording and monitoring health measures, for example, blood pressure; and goal setting and health diaries. | Untethered | Adult patients with hypertension | 16 patients with hypertension | - User themes requiring attention: some difficult to understand terminology, changing relationship with providers, overwhelming and time consuming task of using PHRs, cost of ePHR  - Technology themes mainly reflected on health and technology literacy, patient usability, ease of access, need for additional instructions, and the potential of customizable menus.  - Linkage to the other systems  - Desire to choose those providers which have access to patients' ePHR |
| Nordfeldt et al. (2010) ([9](#_ENREF_9)) | To explore patients’ and parents’ attitudes toward a local Web 2.0 portal tailored to young patients with type 1 diabetes and their parents and opportunities and obstacles to the application of the system | Qualitative | Sweden | A Patient portal called “Diabit” containing specific diabetes-related information and social networking functions such as message boards and blogs and allowing medical prescription renewal, making appointments, sending questions, viewing questions and answers, contact information, photos of staff, and other general information about the local diabetes teams and their services. Used by patients, parents and providers. | Not documented | Children with diabetes | 16 mothers and 3 fathers of sick children, and 5 young patients (11-18 years old) | - The experience of already being in control and having felt secure with the treatment over a long period of time was one reason for limited use of the portal.  - Previous good contact with the practitioners, good continuity over time regarding such relationships, sufficient personal experience with living with diabetes, and perceived long-term success regarding treatment were mentioned as factors that might contribute to a lower perceived need for repetitive use of the portal  - Various unsuccessful user experiences, such as few hits from a specific search or seeing that there had been little activity in the practitioners’ news and updates sections of the portal, could create the perception that the practitioners were not “on their toes"  - Issues with passwords  - Users with particularly negative feelings about their disease and/or health care experiences might not be willing to go through the procedure for logging onto a disease-specific portal. |
| Goel et al. (2011) ([10](#_ENREF_10)) | To identify patient reported barriers to enrollment in a patient portal among patients who did not enroll despite being directly offered this service by their providers | Quantitative | The USA | MyChart, a patient portal allowing a patient to log-on to a secure portal to access personalized health information, including laboratory results and a medication list and sending secure electronic messages to physicians. | Tethered | Adult chronic patients | Chronic patients including diabetes, hypertension, chronic pulmonary disease, coronary artery disease, congestive heart failure, peripheral vascular disease, severe chronic liver disease, renal failure, cancer, and dementia | - Reasons for not enrolling: did not remember discussing the patient portal with their providers (26%), did not attempt enrollment despite remembering a discussion with their providers (63%), and attempted to enroll but did not succeed (11%).  - Reasons for not attempting to enrollment: 60% stated reasons related to lack of information or motivation, 30% reported negative attitudes toward the patient portal, and 8% reported connectivity obstacles  - There were large, but non-signiﬁcant differences in reasons for not attempting enrollment by race; black people mentioned more negative attitudes and connectivity obstacles  - There were large differences in reasons for not attempting enrollment by presence of chronic disease (lack of information/motivation was cited by 55% with chronic disease vs. 71% without chronic disease)  - Additional reasons for not attempting enrollment: 37% said they prefer to call the providers' ofﬁce to discuss health matters rather than communicate electronically and nearly 25% reported they did not feel the internet is a safe way to communicate sensitive health information. |
| Tenforde et al. (2011) ([11](#_ENREF_11)) | To measure the association between use of an advanced electronic medical record-linked PHR and diabetes quality measures in adults with diabetes mellitus (DM). | Quantitative | The USA | MyChart, the Cleveland Clinic’s electronic medical record (EMR)-linked PHR, allowing to access patient's' diagnoses and co-morbidities, laboratory and other test results, along with secure messaging through the PHR with their provider. Patients can also access glucometer readings, a set of diabetes-related health and wellness links, and diabetes specific health reminders (including recommended glycated hemoglobin, urine albumin, and cholesterol testing due dates, recommendation for pneumococcal vaccination, and due dates for diabetic foot and dilated retinal eye exams). | Tethered | Adult diabetes patients | 4,036 diabetes patients | - Compared to non-users, PHR users were younger, had higher incomes and educational attainment, were more likely to be identified as Caucasian, and had better unadjusted and adjusted diabetes quality measure profiles |
| Sarkar et al. (2011) ([12](#_ENREF_12)) | To examine patient use patterns of an innovative internet-based patient portal within a well-characterized large, diverse cohort of adult medically insured patients with diabetes | Quantitative | The USA | An internet-based patient portal allowing to view laboratory test results, email physicians or care team, request medication reﬁlls, and make appointments. | Tethered | Adult diabetes patients | 5671 diabetes patients | -African-American (31%), Latino (34%), and Filipino (32%) participants least likely, and Asian (53%) and White (51%) participants most likely to both request a password for the internet-based patient portal (a marker for internet access and intent to use) and log on to the portal after requesting a password  - Compared to non-Hispanic Caucasians, African-Americans and Latinos had higher odds of never logging on, as did those without an educational degree compared to college graduates  - Age over 70 years was associated with lack of use among the entire cohort  - Compared to those who used the patient portal, nonusers were more likely to have suboptimal control of their diabetes and related risk factors |
| Nielsen et al. (2012) ([13](#_ENREF_13)) | To evaluate the use of a secure internet portal in an academic Multiple Sclerosis (MS) Center | Quantitative | The USA | "PatientSite", a patient internet portal allowing individuals to manage their clinic appointments (making, canceling, or rescheduling with department administrators), request prescription reﬁlls and referrals directly to their physician’s ofﬁce, view their medical records including labs, pathology, and radiology study results, and communicate directly with their provider regarding non-urgent issues through a secure electronic message system. In addition, PatientSite provided web links to helpful health-related information, an account statement for patient medical bills, and technological support to portal users | Tethered | Adult multiple sclerosis patients | 240 multiple sclerosis patients | - Portal users tended to be young patients with minimal physical disability. Independent predictors and barriers of portal use include the number of medications prescribed (OR 1.69, p<0.0001), Caucasian ethnicity (OR 5.04, p<0.007), arm and hand disability (OR 0.23, p<0.01), and impaired vision (OR 0.31, p<0.01).  - Barriers to portal use included being a minority (0.2-fold odds), worse visual acuity (0.31-fold odds) and upper extremity function (0.23-fold odds).  - The number of clinic visits scheduled was greater among portal users compared to non-users (p<0.0001). A trend toward a greater proportion of ‘no-shows’ to clinic was found among portal non-users (4.2%±10.7 vs. 2.1%±7.3, p=0.12). |
| Wagner et al. (2012) ([14](#_ENREF_14)) | To examine the impact of a PHR in patients with hypertension measured by changes in biological outcomes, patient empowerment, patient perception of quality of care, and use of medical services. | Quantitative | The USA | My HealthLink, which provided a secure, comprehensive, electronic record that enables consumers to store PHI. This PHR is ‘’ allowing to view problem lists and information on medications, allergies, and immunizations Core functions also include: secure messaging; access to educational materials; medication interaction checking; recording and monitoring of health measures, for example, BP; and some goal setting and health diaries. | Tethered | Adult patients with hypertension | 443 hypertensive patients | - Younger age, self-reported computer skills, and more positive provider communication ratings were associated with frequency of PHR use vs. no use.  - In multivariate analysis, patients from Family Medicine (versus those from Internal Medicine), those with a greater number of self-reported internet-use items, and higher provider communication scores had signiﬁcantly more frequent PHR use |
| Day and Gu (2012) ([15](#_ENREF_15)) | To find out: what factors influence PHR use? Do perception of ease of use influence patient’s engagement with the software? What is about available software that is considered useful by patients? | Qualitative | New Zealand | PHR linked to their doctor's Practice Management System (PMS) allows viewing laboratory results, diagnosis, immunizations and medications list  Capabilities: interaction patients with their GP, singing patients to system via internet at home and accept electronic invitations | Tethered | Adults chronic patients | Chronic patients (not specified) | - Required computer and health literacy which contribute to being able to effectively use the PHR  - Usability issues (e.g., navigation in general was not intuitive and some PHR functions were not useful)  - Concerns about how PHR-related services are paid for, who pays and under what circumstances and necessity for incentive motivation (e.g., getting a fixed number of free consultations and paying for extra) |
| Emani et al. (2012) ([16](#_ENREF_16)) | To apply a theoretical model, the diffusion of innovation model, to the study of PHRs and conduct an exploratory empirical study on the applicability of the model to the study of perceptions of PHRs | Quantitative | The USA | Patient Gateway, allowing requests for appointments, prescription refills and referrals, access to certain components of the EHR such as laboratory results, and secure messaging with the practice and provider | Tethered | Adult asthma, CHF, hypertension, or diabetes patients | Asthma, CHF, hypertension, or diabetes | - Computer use among non-adopters (75%) was lower than that among PHR users (99%) and rejecters (92%) (P < 0.001). Non-adopters also reported a lower score on personal innovativeness in information technology.  - Innovators were younger than other users and non-adopters (P = 0.001)  - Only 50% of non-adopters had a four-year college degree or more compared to 76% of the innovators, 71% of laggards, and 69% of other users (P = 0.001).  - Only 41% of non-adopters had a total household income of $75,000 or more compared to 75% of laggards, 72% of innovators, and 63% of other users (P < 0.001).  - Non-adopters also differed from innovators and laggards on marital status (47% married; P < 0.001).  - In terms of overall health status, non-adopters reported a lower rating of overall health compared to innovators and laggards, and other users and rejecters reported lower overall health status than innovators. Innovators also reported a smaller number of comorbidities (mean = 2.8) than other users, rejecters, and non-adopters (mean = 3.7).  -The greater the relative advantage, ease of use, and trialability of the PHR, the more patients value the PHR for communicating with their doctor’s office.  - More positive perceptions of privacy and security of information in the PHR are associated with greater perceived value of the PHR. |
| Tom et al. (2012) ([17](#_ENREF_17)) | To examine integrated personal health record use patterns among parents of children with chronic disease and compare ratings of care experiences between integrated PHR users and nonusers. | Quantitative | The USA | In “MyGroupHealth” parents access their child’s account as a proxy through their own account.  Users can viewing: immunizations, test results, after-visit summaries, allergies, medical conditions, health assessments, health plan benefits and medication management  Capabilities: secured messaging and appointment management. | Tethered | Children with chronic disease | Parents of a child with at least one chronic disease (types not specified) | - The top reasons for not using the PHR among nonusers were “too busy”, “forgot login name and/or password”, and “child does not have health care needs”  - Some participants noted that they were not comfortable sharing medical information on the Internet  - Other reasons to not using the PHR: forgot login name and/or password; too difficult to get online access for the PHR; not having access to the high-speed Internet; too difficult to use; no response from system; not sure how to use the Internet  - Preference of other routes of care (e.g. face to face) instead of the PHR |
| Urowitz et al. (2012) ([18](#_ENREF_18)) | To evaluate the experience of patients and providers using an online diabetes management portal for patients. | Qualitative | Canada | A Patient portal which provides access to “Health Library” for diabetes education material (for both patient and providers) and providers access to “Personal Health Records” for allowing patients to consolidate their personal health information including medical and family history, medication details, lifestyle choices, and test results | Tethered | Adults patients with diabetes | Patients with diabetes and their providers i.e., general practitioners (GPs), nurses, nurse practitioners (NPs), dieticians, diabetes educators (DECs), and other clinical staff | - Technical issues regarding usability and discoverability (e.g. access to the internet, difficult data entry, and difficulty in finding items)  - Some patients felt that they were controlling their diabetes well or found that their health measurements had been fairly stable and therefore did not feel the need to enter information.  - Required provider duplicate time and efforts to handle issues related to the PHR use parallel to those in the office time, then viewed it as a tool for patients and other care providers  - Provider concern on overreliance of patients on portals when exacerbations in their condition occur |
| Gordon et al. (2012) ([19](#_ENREF_19)) | To describe the process and outcome of developing and implementing a personal health record for people living with HIV/AIDS | Mixed | The USA | My health profile allowing to access most recent medication lists, test results, information on healthcare providers and payers, viewing an integrated audit log, and enabling the development a continuity of care document | Tethered | Adult | Patients living with HIV | - Potential barriers to use of My Health Proﬁle including functional and computer literacy, privacy and conﬁdentiality concerns, potential reluctance to use technology, and cognitive challenges (e.g., remembering passwords)  - PHR implementation was well matched with the organizational mission and values and priorities related to coordination of care |
| Logue et al. (2012) ([20](#_ENREF_20)) | To describe the results of an exploratory study that  provided an initial test of a theoretical framework to understand an elderly’s decision to participate in  self-directed care | Quantitative | The USA | Without a PHR | Not applicable | Adult chronic condition | Senior adults with chronic conditions | - Older seniors reported less confidence in their ability to use internet-based PHRs and did not perceive that they had the resources in place to use them.  - More men than women agreed that they had access to care, access to the internet, enjoyed computers, saw PHRs to be a better fit with their healthcare needs, and expressed confidence in using the internet to communicate with others and in using an internet-based PHR.  -ethnicity  - Older seniors were less likely to know how to find health resources on the internet and were less interested in observing the use of PHRs.  - Those who knew more about what health resources were available on the internet were more likely to be motivated by incentives to use PHRs.  - Older seniors were less confident in their ability to self-manage their own health. By contrast, older adults did not report less computer access; however, they did  have less access to and familiarity with the internet  - Easier access to care was positively correlated with believing that PHRs offer an advantage over alternative methods, that PHRs were compatible with their current healthcare needs and that PHRs were likely to give them the results that they expected. Those that reported easier access to care also were more likely to express confidence in their abilities to communicate via written language and self-manage their health  - Of the respondents who disagreed or were undecided (relating to the three e-health indicators), 51% (n = 18) reported not having access to a computer and 49% (n = 17) reported not having access to the internet. These results indicate that internet access is a prerequisite to knowing what, where and how to find health resources via the internet  - More females (64%) than males (20%) reported not knowing how to use internet-based PHRs;  - The intention to use PHRs within the next year was positively correlated with the likelihood of accepting incentives to use them. In addition, incentive motivation was positively correlated with an individual’s confidence in using an internet-based PHR and the likelihood that they would choose a provider who uses it  - Many more females  (28%) were worried about privacy compared with males (10%)  - Those who preferred to work together with their healthcare provider as a team were more likely to be motivated to learn new things, know what health resources were available via the internet, believe that using an internet-based PHR would give them the health outcomes they sought, be incentivised to use PHRs, prefer to control who could access their PHR see a fit between their current healthcare needs and PHRs, be interested in trying one  - Positive correlations were also noted between the number of illnesses the person reported and PHRs fitting their current healthcare needs. Respondents with more illnesses were more likely to choose a healthcare provider based on the provider’s use of information from their PHR. Those with multiple healthcare providers were the same people who preferred to manage their own health, intended to use a PHR within the next year, believed that PHRs were compatible with their current healthcare needs and would choose a provider based on the provider’s use of the information from their PHRs |
| Britto et al. (2013) ([21](#_ENREF_21)) | To examine parents' perceptions of the benefits and / or drawbacks of a patient portal for managing their child's chronic illness. | Qualitative | The USA | A secure Internet-based application which integrated to an EHR  Users can viewing: laboratory results, visit history, medication information  Capabilities: secure messaging to health care providers, upload documents and share with health care providers and reminders for laboratory tests and clinic visits. | Tethered | Children with Cystic fibrosis, Diabetes mellitus or Juvenile idiopathic arthritis | Parents of children with cystic fibrosis, diabetes mellitus or juvenile idiopathic arthritis | - A potential concern on the loss of interpersonal contact with providers and some parents' preference for direct communication, particularly when hearing bad medical news  - A concern about not knowing who would receive electronic communications and whether anyone would answer |
| Osborn et al. (2013) ([22](#_ENREF_22)) | To (1) understand who uses an existing patient portal and reasons for use and nonuse, (2) understand how portal users are using a portal to manage their medications, and (3) explore participants’ ideas for improving portal functionality for medication management and adherence support. | Mixed | The USA | MyHealthAtVanderbilt, a patient portal allowed managing medical bills, viewing PHI (eg, vital signs, laboratory results, medication lists, and diagnoses) from their electronic health record (EHR), using secure messaging to communicate with providers and manage medical appointments, and view educational contents | Tethered | Adults diabetes patients type 2 | 75 adults with type 2 diabetes | - Users were more likely than nonusers to be Caucasian/white, have higher incomes, and be privately insured. Users also tended to have more education than nonusers  - Reasons for nonuse included not knowing about the portal, not having access to a computer, or having a family member serve as an online delegate. |
| Ronda et al. (2013) ([23](#_ENREF_23)) | To study the characteristics, the health status, the self-efficacy, the diabetes knowledge, and the treatment satisfaction of patients with diabetes who do and do not have a login for a patient Web portal | Quantitative | The Netherlands | A patient portal allowing users to access their medical records, including the information provided by their healthcare provider during medical consultation, such as physical examination, laboratory results, problem lists, and treatment goals. It also provides access to general diabetes information and an overview of all examinations and diabetes visits that are needed according to guidelines. Patients can upload the glucose levels measured at home and seek contact with their care provider through secured electronic messaging | Tethered | Adults diabetic patients types 1 and 2 | Diabetic patients of 18–85 years old | - The participants with a login were significantly younger compared with those without. Of the participants with a login, 63.1% were male compared with 56.5% of the group without login.  - In Type 1 diabetes: patients with a login were younger and had a higher education level. Following the guidelines, most type 1 diabetes patients were treated by an internist; however, patients without a login were more frequently found to be treated in a general practice.  Type 2 diabetes: patients with a login had been diagnosed with diabetes for a longer time, and used insulin more frequently and also used more other drugs compared to those without a login.  - Patients without a login significantly perceived less diabetes-related distress than patients with login and also had less self-efﬁcacy and lower diabetes knowledge.  - With increasing age, the odds of requesting a login decreased. Also, the odds of requesting a login increased in males, in patients with a higher education level, in patients who speak Dutch ﬂuently, and in patients with a paid job, whereas the odds decreased in patients treated by a primary care physician (vs. an internist) or living alone. |
| Wade-Vuturo et al. (2013) ([24](#_ENREF_24)) | To identify the beneﬁts of and barriers to using secure messaging (SM) within a portal. | Mixed | The USA | A patient portal which called “MyHealthAtVanderbilt (MHAV)”. Users can view EHR data, use secure messaging to communicate with providers, manage medical appointments and bills, and perform other tasks. | Tethered | Adult diabetes patients type 2 | Patients with type 2 diabetes | - Barriers to using SM: (a) preconceived beliefs about technology or rules about SM (e.g., the questionable reliability of the patient portal to facilitate a timely and productive message exchange with their providers, (b) prior negative experiences with SM (e.g., not receiving a response to a patient-initiated message).  - Perceptions of provider endorsement of SM i.e., (a) participants’ assumptions about providers’ willingness to use SM, providers being interrupted by SM, and providers not being reimbursed for SM, (b) providers’ instructions about SM (e.g., participants recounted instances when providers instructed them not to use SM) |
| Lyles et al. (2013) ([25](#_ENREF_25)) | To examine the associations between patient ratings of provider communication or trust with portal use in diabetes patients. | Quantitative | The USA | Patient portal allowing users to view visit summaries, medical history, and/or immunizations/allergies, making appointments, order medication refills, view the results of medical tests, and send or receive secure electronic messages with providers. | Tethered | Adults diabetics patients | Diabetes patients | - There were a signiﬁcant but modest adjusted association between increased trust and being a registered user  - Among patients ≥70 years of age, there was a signiﬁcant association between patient-provider communication and portal use  -There were also signiﬁcant association between trust in provider and race/ethnicity and age. Latino subjects were more likely to be a registered user when there was higher trust in the provider, as were white patients and patients ≥70 of age  - After adjustment, there was a signiﬁcant association between trust in provider and overall secure message use |
| Pai et al. (2013) ([26](#_ENREF_26)) | To determine the experience of, and feedback from, prostate cancer patients using a PHR, while receiving care from a provincial cancer agency. | Mixed | Canada | “Provider”: a web-based integrated with an electronic clinical information system to store and access the medical records of patients with cancer.  With access to laboratory, pathology, imaging, operative, and procedure reports, scheduling and appointment information and medications, secure messaging patient-provider and etc. | Tethered | Adult patients with prostate cancer | Male patients with prostate cancer | - Mixed responses and lack of clarity on who should pay for the PHR, for example, federal government, provincial government, cancer agency (that is, health care providers), donations or charities, private industry, clients (that is, patients), and other. Besides choosing other options, patients felt that the government should help fund the PHR.  - Several operational difficulties with the "Provider" Web site were reported by both patients or the research assistant |
| Martinez et al. (2013) ([27](#_ENREF_27)) | To identify the characteristics of PHR users versus non-users | Quantitative | Argentina | A web-based PHR allowing patients to view laboratory results, diagnosis, preventive information and medications lists and also to communicate with doctors or health care systems (e.g., for massaging system, appointments scheduling or medication delivery, and to get support for self-management) | Tethered | Adult chronic patients | Chronic patients with hypertension, diabetes, dyslipidemia, cerebral vascular disease, coronary artery disease, chronic heart failure, chronic renal failure, peripheral vascular disease, and smokers | - PHR users were younger and women and had at least one disability or chronic condition and had asked for medical assistance during the last year  - The main predictor of PHR use was being a patient asking for medical assistance during the last year, increasing the PHR use by almost 4 times. |
| Luque et al. (2013) ([28](#_ENREF_28)) | To assess barriers and facilitators to use of online PHRs among persons living with human immunodeficiency virus | Mixed | The USA | Using an exemplary PHR | Tethered | Adult | Patients living with human immunodeficiency virus | - Lack of computer or broadband access and also privacy when accessing a portal outside of one’s home were mentioned as important barriers; computer literacy as a barrier but not as an insurmountable one  - Barriers to the use of the Internet cited by respondents were cost (16/90,18%), lack of interest (6/90, 22%) and do not know how to use (5/90, 19%). |
| Byczkowski et al. (2014) ([29](#_ENREF_29)) | To assess parents understanding of the importance of PHR, their concerns for using web-based portals for their children’s diseases | Mixed | The USA | A web based patient portal allowing access laboratory result, medication information, and a child's visit history | Tethered | Children with cystic fibrosis, diabetes mellitus, and juvenile idiopathic arthritis | 530 parents  of children with cystic fibrosis, diabetes mellitus, and juvenile idiopathic arthritis | - 12 percent mentioned that they sometimes saw information in the portal that frightened them, and 11 percent reported that they sometimes see information that they would have preferred to get directly from their provider.  - Requests by parents for easier access to the system and navigation through it, more personalized information according to the child's condition, more medical terminology explanations, and notifications for new lab results |
| Fiks et al. (2014) ([30](#_ENREF_30)) | To design a portal to facilitate shared decision making between families of children with asthma and primary care clinicians based on user-identified criteria and integrated within the EMR | Qualitative | The USA | A Patient portal which called “MyAsthma” and it’s was designed to work within the framework of an existing patient portal, MyChart, and was linked to The children’s Hospital of Philadelphia’s EMR through a Web-based framework | Tethered | Children with asthma | 7 parents of children with asthma and 51 care providers including pediatricians, nurses, and a pharmacist | - Preference for direct communication with physicians  - System interface should be simple and the content be clear  - Provider concern: should be viewed as access to care for chronic condition and not for an acute flare in the condition |
| Sharp et al. (2014) ([31](#_ENREF_31)) | To characterize the knowledge, interest, and attitudes of childhood cancer survivors and their caregivers towards ePHRs. | Qualitative | The USA | Without a specific PHR | Not applicable | Children with cancers | Caregivers of survivors who were <14 years old and also survivors ≥14 years old along with their caregivers when present | - Data security and privacy were the primary concerns expressed by those who had a concern. However, among them, 67% of survivors and 80% of caregivers stated that the concern would not prevent them from using an ePHR. |
| Odlum et al. (2014) ([32](#_ENREF_32)) | To assess the ease of use and usefulness of My Health Profile (MHP) and to identify the actual information needs of MHP-users and perceived information needs of MHP-users and MHP non-users before MHP-plus roll out. | Mixed | The USA | MyHealthProfile, a continuity of care document enabling access to facets of medical records through the internet | Untethered | Adults people living with HIV | People living with HIV | - Problematic issues including incomplete health information in the MHP (e.g., lacking vaccinations and diagnostic test results); and confusing information display in MHP.  - Participants expressed the need for health information to better facilitate provider visits.  - Frustration about how to grant providers access, and whether they know how to use the system |
| Barron et al. (2014) ([33](#_ENREF_33)) | To explore whether older adults with chronic conditions and/or their caregivers demonstrate capacity to use a patient portal, and their perspectives on the experience | Qualitative | The USA | A patient portal enables accessing the P/A/M/I lists, office notes, hospital discharge summaries, and test results | Tethered | Adult chronic obstructive pulmonary disease or congestive heart failure | 14 patients and 19 caregivers | - Usability issues related to unfamiliar medical terms, font and color contrast issues, and poor section labeling |
| Baudendistel et al. (2015) ([34](#_ENREF_34)) | To explore needs and requirements of potential users with regard to the content and function of a patient-controlled personal electronic health record | Qualitative | Germany | Without a PHR | Not applicable | Adult patients with colorectal cancer | Patients with colorectal cancer, health care providers, clinicians, clinical staff in an umbrella company | - Needs and requirements: a structure necessary to facilitate tracking the course of illness and treatment over time for both physicians and patients; highlight important or new information with color or a priority for current issues; include a basic dataset of relevant information that would be crucial for everyone involved in the patients’ health care with manageable volume of information  - The presentation of information should be in a patient assessable, accessible, and comprehensible way.  - Given the fact that several physicians from different health care settings would have PHR access, physicians expressed concerns and uncertainty regarding negative  consequences on professionals’ liability for reacting to patient-added information or commentaries |
| Gartrell et al. (2015) ([35](#_ENREF_35)) | To examine factors associated with ePHR use by nurses  for their own health management | Quantitative | The USA | Different PHRs | Not applicable | Adults chronic patients | 664 sick nurses with chronic conditions in 12 hospitals | - A larger percent of ePHR users had a chronic medical condition and/or were taking a prescribed medication (71%) compared to non-users (65%) (P<0.05)  - A large portion of PHR users used the Internet frequently (several time per day) compared with PHR non-users (P<0.05)  -A larger portion of ePHR users were more aware of health technologies; and nearly 80% of users compared with approximately 50% of nonusers indicated their primary care providers currently used an EHR for care (p<0.01)  - More ePHR nonusers (72%) were concerned about general privacy and security of health information online compared with users (64%, p=0.06)  - Factors associated with ePHR use: being an active healthcare consumer (having a chronic health condition and taking prescribed medications) and having a healthcare provider using a EHR for care, |
| Gee et al. (2015) ([36](#_ENREF_36)) | To learn from chronically ill engaged and educated (e-patient) adults how and why they use PHRs for self-management support and productive patient-provider interactions. | Qualitative | The USA | Different PHRs (Core functions were not documented) | All tethered | Adults chronic patients | 18 chronic patients | - Health and computer literacy issues (e.g., understanding numbers related to their individual health conditions and navigating through the PHR)  - Usability issues (e.g., the use of medical jargons)  - Frustration following seeing incomplete or incorrect profile, medication, and medical history data in the PHR but not being able to intervene through the system.  - Lack of proper or adequate user training on the use of PHR  - Concerns about privacy of personal or banking data and information  - Provider related issues (e.g., request for involving other providers such as pharmacists, therapists, dietitians); lack of clarity about who may see the content of their messages (i.e., their physicians or office staff); providers not using the PHR; and concerns about the provider workload following PHR use  - A lack of interoperability between systems in provider offices and other systems and the resulting frustration related to care coordination between offices that use EHR/PHR systems and those still using paper-based systems. |
| Harrison et al. (2015) ([37](#_ENREF_37)) | To understand perceptions of CKD patients about ePHRs, and describe characteristics associated with their expressed intent to use an ePHR. | Quantitative | Canada | Without a PHR | Not applicable | Adults patients with non-dialysis-dependent CKD | Patients with non-dialysis-dependent CKD | - Patients over the age of 65 were less likely to intend to use an ePHR  - No association between gender or self-perceived health and intent to use the ePHR  - Those with post-secondary education and Internet access were more likely to express their intent to use an ePHR  - 69.8 % of our patient group intended to use an ePHR if it became available  - Patients who did not convey intent to use the ePHR did not report anticipated benefit of ePHR use as often  -The perceived benefits of greater personal involvement in healthcare, access to health information and lab results were associated with expressed intent to use  - Privacy of health records as the most common concern noted regarding ePHR use |
| Nippak et al. (2015) ([38](#_ENREF_38)) | To explore the perceptions of family members regarding the importance of an electronic personal health record to support the care of their loved ones within a long term care facility | Mixed | Canada | MyChart: a secure and private web-based platform that offers self-management tools that are entirely accessed and controlled by patients such as diaries to record their health history, symptoms, and medications, emergency contact information as well it provides access to health education sites and appointment scheduling features | Not documented | Adults elderly chronic patients | Family members of residents residing in a long term care facility | Family members identiﬁed concerns linked to:  - Privacy, conﬁdentiality, and security of the electronic health record information  - The knowledge and understanding e.g., not understanding the information and fearing abnormal values  - Issues related to the use of MyChart on staff operations and their communication exchange with family members such as staff workload and impact on communication between staff and patients |
| Tieu et al. (2015) ([39](#_ENREF_39)) | To explore the barriers and facilitators to use of a patient portal in anticipation of portal implementation in an urban, safety net primary care clinic | Qualitative | The USA | Patient portals in general/without a specific PHR | Not applicable | Adult chronic patients | 11 patients with chronic illness including diabetes and 5 caregivers | - Health and computer literacy challenges (e.g., problems with reading and typing; personal experience with online security breaches/viruses, and distrust of potential security measures; lack of basic computer skills and problems of handling passwords; challenges with the medical terminology and lack of language-appropriate information)  - Concerns about the affordability of the Internet, particularly the cost of mobile data  - Concern about privacy, confidentially and security of patients' health information particularly sensitive diagnoses and medications being online or easily accessible to researchers and industry members  - Concern over technology replacing the healthcare providers and diminishing or interfering with ongoing in-person communication with them |
| Wells et al. (2015) ([40](#_ENREF_40)) | To investigate organizational strategies to promote PHR adoption with a focus on patients with chronic disease. | Mixed | The USA | Different PHRs across the country | Not applicable | Adult chronic patients in general | Chief information officers, directors of e-health services, medical directors, or internists with specialized roles in chronic disease management, quality, primary care, or population health | - The greatest barrier to PHR implementation was perceived to be physician resistance due to concerns about the impact on their workload and on their patients.  - Discussions around how to better reimburse provider interactions by PHR |
| Latulipe et al. (2015) ([41](#_ENREF_41)) | To investigating facilitators and barriers to adoption of patient portals among low-income, older adults in rural and urban populations older adults in rural and urban populations | Qualitative | The USA | A patient portal in general/Without a specific patient portal | Not applicable | Adults chronic patients | 36 chronic patients and 16 caregivers (chosen from low-income, older adult populations across the country) | - Lack of interest in technology in general and portal use (15 out of 36 patient participants) as well, which appeared to be linked to age  - Lacking confidence with respect to technology use especially in older participants (e.g., remembering passwords)  - The most frequent concern noted was that of privacy and security (e.g., leading to for example misuse of information by insurance companies to deny coverage)  - A fear that use of the patient portal could eventually replace face-to-face visits with their healthcare provider and this was seen as a potential negative consequence of signing up  - Stress following reading medical information instead of hearing them  - Unclear from who to get technical assistance in the case of a problem |
| Smith et al. (2015) ([42](#_ENREF_42)) | To document disparities in registration and use of an online patient portal among older adults. | Quantitative | The USA | A patient portal allowing three main options (message a provider, request a prescription reauthorization, and view test results) with additional options including personal health records (monitoring vital statistics [e.g. height, weight, body mass index, body surface area, blood pressure, heart rate, breathing rate, temperature], previous conditions, and current conditions), previous or upcoming appointments, sent and received messages, personal profile, and a help page. | Tethered | Adults with chronic conditions including arthritis, asthma, bronchitis or emphysema, cancer, coronary heart disease, depression, diabetes, heart failure, and hypertension. | 534 older adults with chronic conditions including arthritis, asthma, bronchitis or emphysema, cancer, coronary heart disease, depression, diabetes, heart failure, and hypertension. | - White patients, male gender, college graduates and those with marginal or adequate health literacy were more likely to have registered their patient portal and use its options. |
| Eschler et al. (2016) ([43](#_ENREF_43)) | How do individuals characterize their experiences of and expectations for using asynchronous communication strategies to coordinate health care with clinicians? | Qualitative | The USA | A patient portal allowing to view medical test results, visit summaries, immunization lists, allergy lists, medical condition lists, exchanging secure messaging with providers, ordering medication refills, scheduling an in-person appointment | Tethered | Children with asthma and adults with diabetes | 7 parents of children with asthma and 12 adult diabetes patients | - Failing to track issues following a secure communication with care providers such as a lack of status indicators for unresolved issue; and exposing patients to inconsistent communication patterns such a by either a call or a written note, which confuses patients leading to potential lapses in illness management |
| Schneider et al. (2016) ([44](#_ENREF_44)) | To understand patients’ lived experience with a patient-controlled electronic health record (PCEHR) and how the use of such a technology may lead to patient empowerment | Qualitative | The UK | A patient-controlled electronic health record, called Patients Know Best It allowed patients and clinicians alike to upload, enter, view, and edit various health data (e.g., symptoms, medications, diagnoses, test results, and body measurements). It also provided features such as electronic messaging, video conferencing, and file management. | Untethered | Children with chronic gastrointestinal diseases | 16 parents of sick children and a teenager who was ill as well as 11 clinicians | - Patients' willingness to take power and responsibility for their health through using technology depends heavily on the patient’s coping style and perceived competence, autonomy, and relatedness.  - Failure of the system in its chronic care context to meet the needs of chronic patients and their caregivers who followed the avoidance-oriented coping style towards their chronic condition, which typically involved denial and suppression of their condition and disengagement in chronic care.  - The avoidance-oriented people used the system only when necessary to coordinate care or to communicate with the clinical team, or did not use it at all, when compared to the approach-oriented people who were found to use the system heavily to track symptoms, medication, and food intake and to investigate test results. |
| Hazara and Bhandari (2016) ([45](#_ENREF_45)) | To evaluate the characteristics and experiences of those patients who have registered for renal patient view but were inactive in using it. | Mixed | The UK | A renal patient view that is a secure website to view and monitor lab results, to document and monitor certain health parameters that of interests for their renal care providers e.g., weight, blood pressure, blood glucose and medications. It also includes educational materials | Tethered | Adult renal chronic patients | 69 chronic renal patients | - Main reasons for being in-active were mentioned as difficulties in using computers or passwords (45%) followed by the perception that it did not add anything to the members' existing relationship with their renal team (37%)  - Other reasons: being too busy, getting anxious when seeing results online, difficult website navigation  - No need to use the website due to satisfaction with the routine communication with the renal care team |
| Graetz et al. (2016) ([46](#_ENREF_46)) | To understand whether socio-demographic differences in patient portal use for secure messaging can be explained by differences in internet access and care preferences. | Quantitative | The USA | A patient portal allowing users to view lab test results, order prescription medication reﬁlls, schedule nonurgent primary care visits, view after-visit summaries, and exchange secure electronic messages with their health care providers. | Tethered | Adult patients with asthma, coronary artery disease, congestive heart | 1041 patients aged 18 or older who had at least one of the following chronic conditions of asthma, coronary artery disease, congestive heart failure, diabetes, or hypertension | - Without adjustment for internet access or care preference, patients who were male, older, of Asian or black race/ethnicity, lower income, and with less education were statistically signiﬁcantly less likely to have used the portal to send a secure message than those who were female, younger, of white race/ethnicity, higher income, and higher education (P<0.05).  - Frequency of internet access was associated with higher use of the portal  - Patients who reported a preference for getting care in-person or over the phone instead of online were less likely to report having used the portal to send a secure message (P<0.001) |
| Ryan et al. (2016) ([47](#_ENREF_47)) | To explore the feelings, ideas and expectations of patients and primary care providers concerning both the implementation and the use of patient portals. | Qualitative | Canada | Without a patient portal/patient portal in general | Not applicable | Adult patients with diabetes, hypertension, asthma, obesity, COPD, thyroid condition, hyperlipidemia and cancer. | 7 patients and 4 providers (i.e., two family physicians, one nurse practitioner and one family practice nurse) | - Challenging or problematic issues related to the accessibility of patient portals regarding computer literacy and the cost of portals (especially if patients need to pay), impact on provider workload, the primacy of direct patient-provider relationship, honesty and trust on the data entered by patients, privacy and confidentiality of information, ability of patients to understand and interpret the content of portals |
| Arcury et al. (2017) ([48](#_ENREF_48)) | To determine potentially modifiable factors affecting patient portal utilization by older adults who receive care at clinics that serve low income and ethnically diverse communities. | Quantitative | The USA | The patient portal systems of the urban and rural clinics differed; but included viewing test results, sending a message to doctors or nurses, refilling prescriptions, making or changing an appointment, requesting a referral, finding information about a health issue, and other | Tethered | Adult patients with diabetes, hypertension, dyslipidemia, or cardiovascular disease | 100 patients with diabetes, hypertension, dyslipidemia, or cardiovascular disease | - Patient portal utilization did not differ by participant age or gender  - Poverty level was associated with patient portal utilization: 91% of those below the poverty level, 74.4% of those at 100% to 200 % of the poverty level, and 53.1% of those above 200% of the poverty level had not utilized their patient portal  - Those with greater than a high school education had greater odds of patient portal utilization  . Those who were not currently married had lesser odds of patient portal utilization  - Receiving care at an urban clinic greatly increased the odds of patient portal utilization  -Those with worse health utilized their patient portal more  - More minority participants (90.8%) than white participants (62.5%) had not utilized their patient portal  - Lesser eHealth literacy was associated with patient not utilizing their portal  -Those with access to e-devices and Internet in their homes (33.9% vs 1.2%), who use the Internet at least once a day (47.5% vs 8.6%), and who experience no stress when using a computer (50.0% vs 11.3% who experience at least some stress) were more likely to utilize their patient portal. |
| Sieck et al. (2017) ([49](#_ENREF_49)) | To examined the following research question:  “Within primary care offices with high rates of patient-portal use, what do experienced physician and patient users of the ambulatory portal perceive as the benefits and challenges of portal use in general and secure messaging in particular?” | Qualitative | The USA | MyChart, an interactive patient portal allowed viewing demographics and test and lab results  Capabilities: schedule appointments, request refills and send secure messages to providers. | Tethered | Adult patients with at least one cardiopulmonary condition | 13 Family Medicine providers in the department of Family Medicine and 29 of their patients who had at least one chronic condition. | - Concerns about imposing on the physician and the lack of provider reimbursement for interactions  - Uncertainty shared by both physicians and patients about how the patient should use the messaging function of PHR (e.g., lack of clarity about when to send a secure message; concern about unfocused or insufficient information in the messages, inappropriate message topics, and incorrect use of the secure messaging feature) |
| Cerdan et al. (2017) ([50](#_ENREF_50)) | To gain insight into the experiences of patients with long-term conditions enrolled in an online rehabilitation program using a web portal. | Qualitative | Denmark | The Digital patient booklet, a patient portal for rehabilitation with supportive information and exercise programs for self-management activities | Not documented | Adults patients with heart disease, lymphedema and chronic pulmonary obstructive disease | Patients with heart disease, lymphedema and chronic pulmonary obstructive disease | - Patients preferred personal contact with physicians and physiotherapists  - Technical issues regarding access, content, graphics and sounds, and terminology such as in non-native language  - Negative attitudes towards PHR following negative attitudes to their received routine care and experiences of confusions, misunderstandings, and contradictory messages from providers regarding their diagnosis |
| Tieu et al. (2017) ([51](#_ENREF_51)) | To examine specific usability barriers to patient portal engagement among a diverse group of patients and caregivers. | Mixed | The USA | Web-based PHR with links to online health education library; allows viewing visit summaries, prescribed health education, test results and looking up general health information | Tethered | Adult patients with diabetes, hypertension, asthma or COPD, heart disease, heart failure or chronic kidney disease | Patients with diabetes, hypertension, asthma or COPD, heart disease, heart failure or chronic kidney disease and their caregivers and 2 care providers | - Basic, health, and computer literacy challenges e.g.,  difficulty understanding  non-health as well as medical terms and the interpretation of treatment plans and test results, inexperienced using search bars or uniform resource locators (URLs), difficulty while navigating the portal |
| Williamson et al. (2017) ([52](#_ENREF_52)) | To characterize how young adult survivors and parent proxies of survivors <18 years old use a PHR | Quantitative | The USA | SurvivorLink, a web-based PHR allowing users to upload and store important health documents and electronically share these documents with their providers independent of institutional or practice specific electronic medical records systems | Stand alone | Pediatric cancer patients | Patients with cancer and their parents | - Black PHR registrants were significantly less likely to use the SurvivorLink in a meaningful way  - Young adult registrants (>18 years old) or those who transitioned during the observation period were significantly more likely to use SurvivorLink in a meaningful way compared to those < 18 (with their proxy parent users). |
| Peremislov (2017) ([53](#_ENREF_53)) | To explore electronic communication (e-communication or e-message encounter) between patients with type 2 diabetes and their providers within the patient portal. | Qualitative | The USA | A patient portal allowing patient-provider e-communication (no further details were documented) | Tethered | Adult type 2 diabetes patients | Patients with type 2 diabetes | - Of 71 e-communications initiated by providers, 49.2 % were from primary care physician (PCP) staff, 30.9% from PCP, 14.1% from care coordinators, 2.8% from pharmacists, 1.4% from diabetes clinic staff and 1.4% from specialty care team members |
| Price-Haywood et al. (2017) ([54](#_ENREF_54)) | To examined the relationship between health literacy, portal use status, and interest in using websites or smartphone applications for tracking health information and to identify speciﬁc facilitators and barriers to use the portal. | Quantitative | The USA | *The patient portal of Epic systems called “MyOchsner” allowing patients to securely schedule/cancel non-urgent appointments, request medication refills, send and receive secure messages, view/download their health records, and access medical tools (e.g., wireless or patient-entered flow sheet data) | Tethered | Adult patients with hypertension and/or diabetes | 247 patients with hypertension and/or diabetes | - Despite high rates of having access to computers, cell phones, and an Internet connection (>70%), portal nonusers most frequently cited preference for phone communication as the most common reason for not using the portal (75%).  - Compared to nonusers, a higher proportion of users rated portal features useful.  - e-health scores were positively associated with higher education and negatively associated with age. The odds of portal usage increased with total e-health score and decreased among black patients. The odds of being interested in using websites/smartphone apps increased with total e-health score  - Portal nonusers mostly expressed concerns about online security of their information, lack of personalization in using technology, lack of resources, desire for skills or technical support to navigate computers and/or the Internet, and simply not seeing the need for or value of using the portal to manage their health.  - There were concerns among users about computer literacy, the cumbersome nature of logging into portal accounts (e.g., remembering passwords, multiple accounts for patients in the same household), lack of technical support, and variations in provider availability for online appointment scheduling and response times to medical messages  - Patient Research Advisory Board identified a lack of a clear tangible incentives for using the portal as a supplement to the traditional provider-patient relationship as a major area of concern for portal nonusers |
| Price-Haywood et al. (2018) ([55](#_ENREF_55)) | To examine whether the intensity of bidirectional secure portal messaging is associated with improved clinical outcomes. | Quantitative | The USA | *The patient portal of Epic systems called “MyOchsner” allowing patients to securely schedule/cancel non-urgent appointments, request medication refills, send and receive secure messages, view/download their health records, and access medical tools (e.g., wireless or patient-entered flow sheet data) | Tethered | Adult patients with hypertension or diabetes | Patients with hypertension or diabetes | - A higher proportion of patients who were age 50 years and older, female, white non-Hispanic, and with co-morbid diabetes and hypertension had higher frequency and intensity of medical advice messaging  - Compared to portal nonusers, portal users were younger, and a higher proportion were female, lived in zip code regions with higher average household incomes, and were commercially insured. Among portal users there were a lower proportion of black non-Hispanic patients, and lower average Charlson comorbidity scores |
| Ali et al. (2018) ([56](#_ENREF_56)) | To identify task-technology fit problems and usability challenges in the novel portal, recommend solutions, and to evaluate whether the recommended design changes improved usability | Mixed | The USA | "myNYP" (New York Presbyterian), an electronic patient portal providing patients with inpatient data such as laboratory results, procedures, and care instructions after their hospital discharge | Tethered | Adults chronic patients | 23 participants which consisted of patients with chronic conditions including types I and II diabetes, and cancer, and also caregivers caring for family members with conditions such as ulcerative colitis and thalassemia | - A number of usability barriers (e.g., failure to use users’ language and insufficient guidance on the portal)  - Mismatch between users, tasks and technology (e.g., lack of a very concrete understanding of health information management tasks  - Problems of consolidating data and medical records scattered across multiple doctors and sharing them |
| van den Heuvel et al. (2018) ([57](#_ENREF_57)) | Primary objectives:  To test the feasibility of a PHR for bipolar patients  To evaluate the user experiences of persons with bipolar disorders (BD) involving informal caregivers, and clinicians.  The secondary objective:  To examine changes in quality of life, empowerment, symptom reduction, changes in mood and activity, and illness burden and severity. | Quantitative | The Netherlands | A Web-based online personal health record  Allowing to view medical record, medication, treatment, and medical passport, laboratory results and reports, mood chart, general information about the features of BD  Capabilities: a personal messages module to communicate with the appointed clinician and a personal crisis plan from interpretation of mood chart | Untethered | Adult bipolar disorder patients | 66 patients with diagnosis of bipolar disorders and eleven clinicians (e.g., psychiatrists, advanced nurse practitioners, and community psychiatric nurses) | - Over a third of the clinicians favored direct telephone contact instead of communication through PHR due to concern that electronic communications distorted open communication and decreased the responsibility of the participant with BD to get in contact for appropriate help  - From those patient participants who not responded at study's endpoint, 81.5% gave the following reasons for dropping-out: the PHRBD was too much work, they did not perceive the added value, were too busy, and felt too confronted when monitoring the course of their illness  - Some clinicians (32.1%) disagreed with informal caregiver access to PHR due to concerns about impairing the privacy of the patient, might be patronizing, and might complicate communication between partners about the illness at a premature stage.  - A lack of compatibility with existing hospital electronic medical record systems by using the messages function (e.g., need to copy and paste reports from the records system into the PHR-BD and vice versa, which was considered too time-consuming and unfeasible for daily practice) |
| Latulipe et al. (2018) ([58](#_ENREF_58)) | To examine how older adult patients perceive the benefits and risks of proxy patient portal access by their caregivers. | Qualitative | The USA | A patient portal (no details available) | Not documented | Adult patients with diabetes, hypertension, dyslipidemia, or cardiovascular disease | 10 patients with diabetes, hypertension, dyslipidemia, or cardiovascular disease | - Concerns about the privacy of their information when a stigmatized condition existed or the confidentially of their financial information |
| Nahm et al. (2018) ([59](#_ENREF_59)) | To examine the current state of older chronic patients'’ patient portal use and their experiences with patient portal training | Mixed | The USA | At least 38 different patient portals across the country | Not applicable | Adult patients with at least one chronic disease including hypertension, arthritis, depression, and others | Patient with at least one chronic disease including hypertension, arthritis, depression, and others | - Participants’ level of knowledge and self-efficacy for PPs were relatively low with an average PP knowledge of 5.2 ± 1.7 and the mean self-efficacy for PP use of 27.1 ± 11.9  - Having multiple portals from multiple providers and not remembering which one was from which provider  - Participants’ perceived usability of their portals (primary if they had multiple) was low, with a mean of 28.7 |
| Powell and Myers (2018) ([60](#_ENREF_60)) | To explore how patients are introduced to and learn about portals and how patients and providers perceive the usefulness of a portal in the context of chronic illness self-management. | Qualitative | The USA | Web based electronic patient portals in general | Not applicable | Adult patients with multiple chronic conditions (diabetes, hypertension, heart disease, or coronary artery disease) | 9 patients and 7 healthcare providers | - Difficulty accessing the portal due to passwords, computer, or server problems identified as a barrier by both patients and providers  - Unavailable functions such as correcting errors in records or changing the preferred pharmacy  - Many patients and providers described their preference for interacting with a person rather than via the portal  - A number of provider-specific barriers with three subcategories: lack of time, payment concerns, and regulatory barriers  - Multiple providers mentioned the need for payment reform, specifically capitated payments, so that providers could be compensated for their work via the portal. |

Abbreviations: personal health records (PHR), electronic medical record (EMR), electronic health record (EHR), chronic kidney disease (CKD), Odd Ratio (OR) chronic obstructive pulmonary disease (COPD), bipolar disorders (BD), human immunodeficiency virus (HIV), my health profile (MGP), the United States of America (the USA), the United Kingdom (the UK),

*data was completed using the authors' another publication i.e., "Primary Care Practice Reengineering and Associations With Patient Portal Use, Service Utilization, and Disease Control Among Patients With Hypertension and/or Diabetes"; Ochsner J. 2017 Spring; 17(1): 103–111.

References:

1. Lober WB, Zierler B, Herbaugh A, Shinstrom SE, Stolyar A, Kim EH, et al. Barriers to the use of a personal health record by an elderly population. AMIA Annual Symposium proceedings AMIA Symposium. 2006:514-8. PubMed PMID: 17238394. Pubmed Central PMCID: 1839577.

2. Hess R, Bryce CL, Paone S, Fischer G, McTigue KM, Olshansky E, et al. Exploring challenges and potentials of personal health records in diabetes self-management: implementation and initial assessment. Telemedicine journal and e-health : the official journal of the American Telemedicine Association. 2007 Oct;13(5):509-17. PubMed PMID: 17999613. Epub 2007/11/15. eng.

3. Zickmund SL, Hess R, Bryce CL, McTigue K, Olshansky E, Fitzgerald K, et al. Interest in the use of computerized patient portals: role of the provider-patient relationship. Journal of general internal medicine. 2008 Jan;23 Suppl 1:20-6. PubMed PMID: 18095039. Pubmed Central PMCID: PMC2338160. Epub 2008/01/10. eng.

4. Britto MT, Jimison HB, Munafo JK, Wissman J, Rogers ML, Hersh W. Usability testing finds problems for novice users of pediatric portals. Journal of the American Medical Informatics Association : JAMIA. 2009 Sep-Oct;16(5):660-9. PubMed PMID: 19567793. Pubmed Central PMCID: PMC2744717. Epub 2009/07/02. eng.

5. Kim EH, Stolyar A, Lober WB, Herbaugh AL, Shinstrom SE, Zierler BK, et al. Challenges to using an electronic personal health record by a low-income elderly population. Journal of medical Internet research. 2009 Oct 27;11(4):e44. PubMed PMID: 19861298. Pubmed Central PMCID: 2802566.

6. Sarkar U, Karter AJ, Liu JY, Adler NE, Nguyen R, Lopez A, et al. The literacy divide: health literacy and the use of an internet-based patient portal in an integrated health system-results from the diabetes study of northern California (DISTANCE). Journal of health communication. 2010;15 Suppl 2:183-96. PubMed PMID: 20845203. Pubmed Central PMCID: PMC3014858. Epub 2010/09/29. eng.

7. Weppner WG, Ralston JD, Koepsell TD, Grothaus LC, Reid RJ, Jordan L, et al. Use of a shared medical record with secure messaging by older patients with diabetes. Diabetes care. 2010 Nov;33(11):2314-9. PubMed PMID: 20739686. Pubmed Central PMCID: PMC2963486. Epub 2010/08/27. eng.

8. Wagner PJ, Howard SM, Bentley DR, Seol YH, Sodomka P. Incorporating patient perspectives into the personal health record: implications for care and caring. Perspectives in health information management. 2010 Oct 1;7:1e. PubMed PMID: 21063546. Pubmed Central PMCID: 2966356.

9. Nordfeldt S, Hanberger L, Bertero C. Patient and parent views on a Web 2.0 Diabetes Portal--the management tool, the generator, and the gatekeeper: qualitative study. Journal of medical Internet research. 2010 May 28;12(2):e17. PubMed PMID: 20511179. Pubmed Central PMCID: PMC2956228. Epub 2010/06/01. eng.

10. Goel MS, Brown TL, Williams A, Cooper AJ, Hasnain-Wynia R, Baker DW. Patient reported barriers to enrolling in a patient portal. Journal of the American Medical Informatics Association : JAMIA. 2011 Dec;18 Suppl 1:i8-12. PubMed PMID: 22071530. Pubmed Central PMCID: PMC3241181. Epub 2011/11/11. eng.

11. Tenforde M, Nowacki A, Jain A, Hickner J. The association between personal health record use and diabetes quality measures. Journal of general internal medicine. 2012 Apr;27(4):420-4. PubMed PMID: 22005937. Pubmed Central PMCID: 3304034.

12. Sarkar U, Karter AJ, Liu JY, Adler NE, Nguyen R, Lopez A, et al. Social disparities in internet patient portal use in diabetes: evidence that the digital divide extends beyond access. Journal of the American Medical Informatics Association : JAMIA. 2011 May 1;18(3):318-21. PubMed PMID: 21262921. Pubmed Central PMCID: 3078675.

13. Nielsen AS, Halamka JD, Kinkel RP. Internet portal use in an academic multiple sclerosis center. Journal of the American Medical Informatics Association : JAMIA. 2012 Jan-Feb;19(1):128-33. PubMed PMID: 21571744. Pubmed Central PMCID: 3240754.

14. Wagner PJ, Dias J, Howard S, Kintziger KW, Hudson MF, Seol YH, et al. Personal health records and hypertension control: a randomized trial. Journal of the American Medical Informatics Association : JAMIA. 2012 Jul-Aug;19(4):626-34. PubMed PMID: 22234404. Pubmed Central PMCID: 3384099.

15. Day K, Gu Y. Influencing factors for adopting personal health record (PHR). Studies in health technology and informatics. 2012;178:39-44. PubMed PMID: 22797017. Epub 2012/07/17. eng.

16. Emani S, Yamin CK, Peters E, Karson AS, Lipsitz SR, Wald JS, et al. Patient perceptions of a personal health record: a test of the diffusion of innovation model. Journal of medical Internet research. 2012 Nov 5;14(6):e150. PubMed PMID: 23128775. Pubmed Central PMCID: 3517342.

17. Tom JO, Mangione-Smith R, Solomon C, Grossman DC. Integrated personal health record use: association with parent-reported care experiences. Pediatrics. 2012 Jul;130(1):e183-90. PubMed PMID: 22689872. Epub 2012/06/13. eng.

18. Urowitz S, Wiljer D, Dupak K, Kuehner Z, Leonard K, Lovrics E, et al. Improving diabetes management with a patient portal: a qualitative study of diabetes self-management portal. Journal of medical Internet research. 2012 Nov 30;14(6):e158. PubMed PMID: 23195925. Pubmed Central PMCID: PMC3510725. Epub 2012/12/01. eng.

19. Gordon P, Camhi E, Hesse R, Odlum M, Schnall R, Rodriguez M, et al. Processes and outcomes of developing a continuity of care document for use as a personal health record by people living with HIV/AIDS in New York City. International journal of medical informatics. 2012 Oct;81(10):e63-73. PubMed PMID: 22841825. Pubmed Central PMCID: 4447096.

20. Logue MD, Effken JA. An exploratory study of the personal health records adoption model in the older adult with chronic illness. Informatics in primary care. 2012;20(3):151-69. PubMed PMID: 23710840. Epub 2012/01/01. eng.

21. Britto MT, Hesse EA, Kamdar OJ, Munafo JK. Parents' perceptions of a patient portal for managing their child's chronic illness. The Journal of pediatrics. 2013 Jul;163(1):280-1.e1-2. PubMed PMID: 23541773. Epub 2013/04/02. eng.

22. Osborn CY, Mayberry LS, Wallston KA, Johnson KB, Elasy TA. Understanding patient portal use: implications for medication management. Journal of medical Internet research. 2013 Jul 3;15(7):e133. PubMed PMID: 23823974. Pubmed Central PMCID: 3713921.

23. Ronda MC, Dijkhorst-Oei LT, Gorter KJ, Beulens JW, Rutten GE. Differences between diabetes patients who are interested or not in the use of a patient Web portal. Diabetes technology & therapeutics. 2013 Jul;15(7):556-63. PubMed PMID: 23777369. Pubmed Central PMCID: PMC3709590. Epub 2013/06/20. eng.

24. Wade-Vuturo AE, Mayberry LS, Osborn CY. Secure messaging and diabetes management: experiences and perspectives of patient portal users. Journal of the American Medical Informatics Association : JAMIA. 2013 May 1;20(3):519-25. PubMed PMID: 23242764. Pubmed Central PMCID: PMC3628058. Epub 2012/12/18. eng.

25. Lyles CR, Sarkar U, Ralston JD, Adler N, Schillinger D, Moffet HH, et al. Patient-provider communication and trust in relation to use of an online patient portal among diabetes patients: The Diabetes and Aging Study. Journal of the American Medical Informatics Association : JAMIA. 2013 Nov-Dec;20(6):1128-31. PubMed PMID: 23676243. Pubmed Central PMCID: 3822118.

26. Pai HH, Lau F, Barnett J, Jones S. Meeting the health information needs of prostate cancer patients using personal health records. Current oncology (Toronto, Ont). 2013 Dec;20(6):e561-9. PubMed PMID: 24311957. Pubmed Central PMCID: PMC3851353. Epub 2013/12/07. eng.

27. Martinez M, Baum A, Gomez Saldano AM, Gomez A, Luna D, Gonzalez Bernaldo de Quiros F. Predictive variables of the use of personal health record: the Hospital Italiano de Buenos Aires study. Studies in health technology and informatics. 2013;192:1171. PubMed PMID: 23920945. Epub 2013/08/08. eng.

28. Luque AE, van Keken A, Winters P, Keefer MC, Sanders M, Fiscella K. Barriers and Facilitators of Online Patient Portals to Personal Health Records Among Persons Living With HIV: Formative Research. JMIR research protocols. 2013 Jan 22;2(1):e8. PubMed PMID: 23612564. Pubmed Central PMCID: 3628162.

29. Byczkowski TL, Munafo JK, Britto MT. Family perceptions of the usability and value of chronic disease web-based patient portals. Health informatics journal. 2014 Jun;20(2):151-62. PubMed PMID: 24056751. Epub 2013/09/24. eng.

30. Fiks AGMDM, Mayne SMHS, Karavite DJMSI, DeBartolo EBS, Grundmeier RWMD. A Shared e-Decision Support Portal for Pediatric Asthma. [Article]: Journal of Ambulatory Care Management April/June 2014;37(2):120-126; 2014.

31. Sharp LK, Carvalho P, Southward M, Schmidt ML, Jabine LN, Stolley MR, et al. Electronic Personal Health Records for Childhood Cancer Survivors: An Exploratory Study. Journal of adolescent and young adult oncology. 2014 Sep 1;3(3):117-22. PubMed PMID: 25276495. Pubmed Central PMCID: PMC4171113. Epub 2014/10/03. eng.

32. Odlum M, Gordon P, Camhi E, Valdez E, Bakken S. Exploring factors related to the adoption and acceptance of an internet-based electronic personal health management tool (EPHMT) in a low income, special needs population of people living with HIV and AIDS in New York City. Studies in health technology and informatics. 2014;201:145-52. PubMed PMID: 24943537. Epub 2014/06/20. eng.

33. Barron J, Bedra M, Wood J, Finkelstein J. Exploring three perspectives on feasibility of a patient portal for older adults. Studies in health technology and informatics. 2014;202:181-4. PubMed PMID: 25000046. Epub 2014/07/08. eng.

34. Baudendistel I, Winkler E, Kamradt M, Langst G, Eckrich F, Heinze O, et al. Personal electronic health records: understanding user requirements and needs in chronic cancer care. Journal of medical Internet research. 2015 May 21;17(5):e121. PubMed PMID: 25998006. Pubmed Central PMCID: PMC4468571. Epub 2015/05/23. eng.

35. Gartrell K, Storr CL, Trinkoff AM, Wilson ML, Gurses AP. Electronic personal health record use among registered nurses. Nursing outlook. 2015 May-Jun;63(3):278-87. PubMed PMID: 25982768. Pubmed Central PMCID: PMC4438260. Epub 2015/05/20. eng.

36. Gee PM, Paterniti DA, Ward D, Soederberg Miller LM. e-Patients Perceptions of Using Personal Health Records for Self-management Support of Chronic Illness. Computers, informatics, nursing : CIN. 2015 Jun;33(6):229-37. PubMed PMID: 25899440. Epub 2015/04/23. eng.

37. Harrison TG, Wick J, Ahmed SB, Jun M, Manns BJ, Quinn RR, et al. Patients with chronic kidney disease and their intent to use electronic personal health records. Canadian journal of kidney health and disease. 2015;2:23. PubMed PMID: 26075082. Pubmed Central PMCID: PMC4465011. Epub 2015/06/16. eng.

38. Nippak PMD, Isaac WW, Geertsen A, Ikeda-Douglas CJ. Family attitudes towards an electronic personal health record in a long term care facility. Journal of Hospital Administration. 2015;4(3):9-19.

39. Tieu L, Sarkar U, Schillinger D, Ralston JD, Ratanawongsa N, Pasick R, et al. Barriers and Facilitators to Online Portal Use Among Patients and Caregivers in a Safety Net Health Care System: A Qualitative Study. Journal of medical Internet research. 2015 Dec 3;17(12):e275. PubMed PMID: 26681155. Pubmed Central PMCID: PMC4704882. Epub 2015/12/19. eng.

40. Wells S, Rozenblum R, Park A, Dunn M, Bates DW. Organizational strategies for promoting patient and provider uptake of personal health records. Journal of the American Medical Informatics Association : JAMIA. 2015 Jan;22(1):213-22. PubMed PMID: 25326601. Pubmed Central PMCID: PMC4433381. Epub 2014/10/19. eng.

41. Latulipe C, Gatto A, Nguyen HT, Miller DP, Quandt SA, Bertoni AG, et al. Design Considerations for Patient Portal Adoption by Low-Income, Older Adults. Proceedings of the SIGCHI conference on human factors in computing systems CHI Conference. 2015 Apr;2015:3859-68. PubMed PMID: 27077140. Pubmed Central PMCID: PMC4827765. Epub 2016/04/15. eng.

42. Smith SG, O'Conor R, Aitken W, Curtis LM, Wolf MS, Goel MS. Disparities in registration and use of an online patient portal among older adults: findings from the LitCog cohort. Journal of the American Medical Informatics Association : JAMIA. 2015 Jul;22(4):888-95. PubMed PMID: 25914099. Pubmed Central PMCID: PMC4810779. Epub 2015/04/29. eng.

43. Eschler J, Meas PL, Lozano P, McClure JB, Ralston JD, Pratt W. Integrating the patient portal into the health management work ecosystem: user acceptance of a novel prototype. AMIA Annual Symposium proceedings AMIA Symposium. 2016;2016:541-50. PubMed PMID: 28269850. Pubmed Central PMCID: PMC5333335. Epub 2017/03/09. eng.

44. Schneider H, Hill S, Blandford A. Patients Know Best: Qualitative Study on How Families Use Patient-Controlled Personal Health Records. Journal of medical Internet research. 2016 Feb 24;18(2):e43. PubMed PMID: 26912201. Pubmed Central PMCID: PMC4785240. Epub 2016/02/26. eng.

45. Hazara AM, Bhandari S. Barriers to patient participation in a self-management and education website Renal PatientView: A questionnaire-based study of inactive users. International journal of medical informatics. 2016 Mar;87:10-4. PubMed PMID: 26806707. Epub 2016/01/26. eng.

46. Graetz I, Gordon N, Fung V, Hamity C, Reed ME. The Digital Divide and Patient Portals: Internet Access Explained Differences in Patient Portal Use for Secure Messaging by Age, Race, and Income. Medical care. 2016 Aug;54(8):772-9. PubMed PMID: 27314262. Epub 2016/06/18. eng.

47. Ryan BL, Brown JB, Terry A, Cejic S, Stewart M, Thind A. Implementing and Using a Patient Portal: A qualitative exploration of patient and provider perspectives on engaging patients. Journal of innovation in health informatics. 2016 Jul 4;23(2):848. PubMed PMID: 27869582. Epub 2016/11/22. eng.

48. Arcury TA, Quandt SA, Sandberg JC, Miller DP, Jr., Latulipe C, Leng X, et al. Patient Portal Utilization Among Ethnically Diverse Low Income Older Adults: Observational Study. JMIR medical informatics. 2017 Nov 14;5(4):e47. PubMed PMID: 29138129. Pubmed Central PMCID: PMC5705857. Epub 2017/11/16. eng.

49. Sieck CJ, Hefner JL, Schnierle J, Florian H, Agarwal A, Rundell K, et al. The Rules of Engagement: Perspectives on Secure Messaging From Experienced Ambulatory Patient Portal Users. JMIR medical informatics. 2017 Jul 4;5(3):e13. PubMed PMID: 28676467. Pubmed Central PMCID: PMC5516097. Epub 2017/07/06. eng.

50. Cerdan J, Catalan-Matamoros D, Berg SW. Online communication in a rehabilitation setting: Experiences of patients with chronic conditions using a web portal in Denmark. Patient education and counseling. 2017 Dec;100(12):2283-9. PubMed PMID: 28698033. Epub 2017/07/13. eng.

51. Tieu L, Schillinger D, Sarkar U, Hoskote M, Hahn KJ, Ratanawongsa N, et al. Online patient websites for electronic health record access among vulnerable populations: portals to nowhere? Journal of the American Medical Informatics Association : JAMIA. 2017 Apr 1;24(e1):e47-e54. PubMed PMID: 27402138. Pubmed Central PMCID: PMC6080722. Epub 2016/07/13. eng.

52. Williamson RS, Cherven BO, Gilleland Marchak J, Edwards P, Palgon M, Escoffery C, et al. Meaningful Use of an Electronic Personal Health Record (ePHR) among Pediatric Cancer Survivors. Applied clinical informatics. 2017 Mar 15;8(1):250-64. PubMed PMID: 28293684. Pubmed Central PMCID: PMC5373768. Epub 2017/03/16. eng.

53. Peremislov D. Patient Use of the Electronic Communication Portal in Management of Type 2 Diabetes. Computers, informatics, nursing : CIN. 2017 Sep;35(9):473-82. PubMed PMID: 28323648. Epub 2017/03/23. eng.

54. Price-Haywood EG, Harden-Barrios J, Ulep R, Luo Q. eHealth Literacy: Patient Engagement in Identifying Strategies to Encourage Use of Patient Portals Among Older Adults. Population health management. 2017 Dec;20(6):486-94. PubMed PMID: 28384076. Epub 2017/04/07. eng.

55. Price-Haywood EG, Luo Q, Monlezun D. Dose effect of patient-care team communication via secure portal messaging on glucose and blood pressure control. Journal of the American Medical Informatics Association : JAMIA. 2018 Jun 1;25(6):702-8. PubMed PMID: 29444256. Epub 2018/02/15. eng.

56. Ali SB, Romero J, Morrison K, Hafeez B, Ancker JS. Focus Section Health IT Usability: Applying a Task-Technology Fit Model to Adapt an Electronic Patient Portal for Patient Work. Applied clinical informatics. 2018 Jan;9(1):174-84. PubMed PMID: 29539648. Pubmed Central PMCID: PMC5851788. Epub 2018/03/15. eng.

57. van den Heuvel SCGH, Meije D, Regeer EJ, Sinnema H, Riemersma RF, Kupka RW. The user experiences and clinical outcomes of an online personal health record to support self-management of bipolar disorder: A pretest-posttest pilot study. Journal of Affective Disorders. 2018 2018/10/01/;238:261-8.

58. Latulipe C, Quandt SA, Melius KA, Bertoni A, Miller DP, Jr., Smith D, et al. Insights Into Older Adult Patient Concerns Around the Caregiver Proxy Portal Use: Qualitative Interview Study. Journal of medical Internet research. 2018 Nov 2;20(11):e10524. PubMed PMID: 30389654. Pubmed Central PMCID: PMC6240158. Epub 2018/11/06. eng.

59. Nahm ES, Zhu S, Bellantoni M, Keldsen L, Charters K, Russomanno V, et al. Patient Portal Use Among Older Adults: What Is Really Happening Nationwide? Journal of applied gerontology : the official journal of the Southern Gerontological Society. 2018 May 1:733464818776125. PubMed PMID: 29779422. Epub 2018/05/22. eng.

60. Electronic Patient Portals: Patient and Provider Perceptions [Internet]. 2018 [cited 15th, April 2008]. Available from: <http://www.himss.org/ojni>.

1. In the chronological order of publication year [↑](#footnote-ref-1)
